# OSI Stack Redesign for Quantum Networks: Requirements, Technologies, Challenges, and Future Directions

**DOI:** 10.3390/s26185696

**Published:** 2026-09-08

**Authors:** Shakil Ahmed, Yehia Osman, Luke Cue, Ibrahim Almazyad, Nasser S. Albalawi, Muhammad Kamran Saeed, Ashfaq Khokhar

**Affiliations:** 1 Department of Computer Science, Grand Valley State University, Allendale, MI 49401, USA; 2 Department of Electrical and Computer Engineering, Iowa State University, Ames, IA 50011, USA; yosman@iastate.edu (Y.O.);; 3 Department of Computer Engineering, Qassim University, Buraydah 52571, Saudi Arabia; i.almazyad@qu.edu.sa; 4 Department of Computer Science, Northern Border University, Arar 73213, Saudi Arabia

**Keywords:** quantum and cognitive networking, OSI, post-quantum cryptography, quantum-classical integration

## Abstract

Quantum communication is emerging as a foundation for next-generation networks, offering unprecedented capabilities in security, entanglement-based connectivity, and distributed computation. However, the classical Open Systems Interconnection (OSI) model, designed for deterministic, error-tolerant systems, is incompatible with quantum phenomena such as decoherence, probabilistic entanglement, and the no-cloning theorem. This paper surveys and redefines the OSI model for quantum networking in the context of 7G systems. We propose a Quantum-Converged OSI stack by extending the classical seven-layer model with two additional layers: (i) Layer 0, the Quantum Substrate, responsible for entanglement management, coherence preservation, and teleportation; and (ii) Layer 8, the Cognitive Intent Plane, which enables AI- and QML-driven orchestration. The survey synthesizes over 150 research works published between 2018 and 2025, classifying them by OSI layer, enabling technologies (e.g., Quantum Key Distribution, Quantum Error Correction, and Post-Quantum Cryptography), and application domains such as satellite quantum links, quantum IoT, and federated edge systems. We further provide a taxonomy of cross-layer enablers and discuss simulation tools, including NetSquid, QuNetSim, and QuISP. Finally, an evaluation framework with quantum-native metrics, such as entropy throughput, coherence latency, and entanglement fidelity, is introduced, along with open challenges for programmable stacks, digital twins, and AI-defined quantum agents. The specific and novel contribution of this work is a Quantum-Converged OSI stack that extends the classical seven-layer model with two additional layers: Layer 0, the Quantum Substrate, responsible for entanglement management, coherence preservation, and teleportation; and Layer 8, the Cognitive Intent Plane, which enables AI- and QML-driven orchestration. Unlike prior technology-centric surveys, the proposed framework classifies over 150 research works by OSI layer, maps enabling technologies (QKD, QEC, PQC) and application domains (satellite quantum links, quantum IoT, federated edge systems) to their functional layers, and introduces a quantum-native evaluation framework based on entropy throughput, coherence latency, and entanglement fidelity. This layer-resolved synthesis, together with the formal definition of cross-layer quantum-native metrics, constitutes the principal novelty distinguishing this survey from existing quantum-networking reviews.

## 1. Introduction

**Figure 1:**
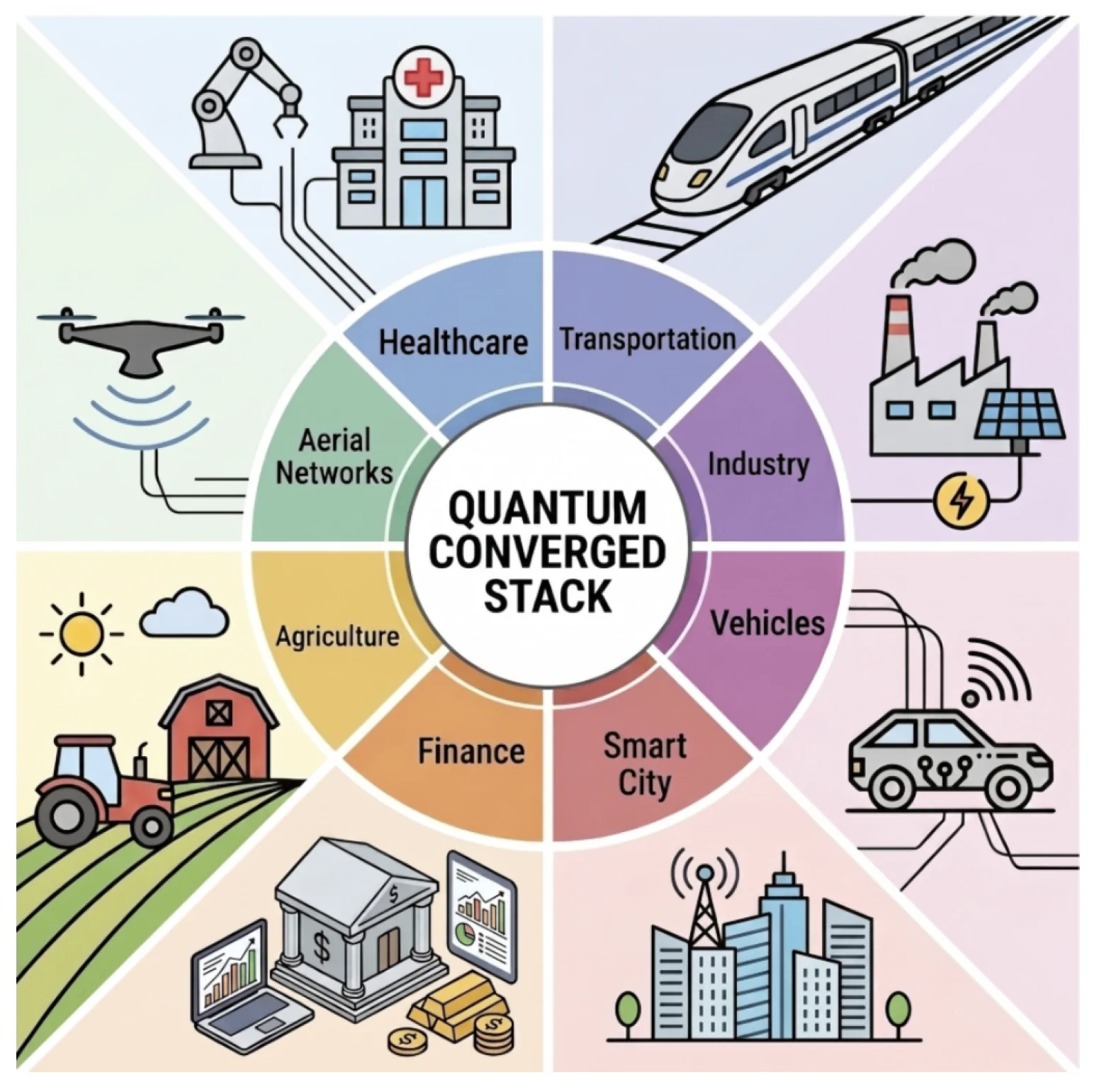
Relationship between application domains and shared quantum-network infrastructure. Vertical sectors represent domain-specific use cases, while the horizontal integration layer represents common communication, computing, and security services built upon quantum technologies.

Quantum communication is rapidly emerging as a foundational enabler for future wireless systems, particularly in the context of sixth-generation (6G) and seventh-generation (7G) networks. While classical networks rely on deterministic signal transmission and replication, quantum communication relies on principles such as superposition, entanglement, and the no-cloning theorem, introducing fundamentally new constraints and capabilities. These include ultra-secure transmission through Quantum Key Distribution (QKD), quantum teleportation for information exchange, and non-local correlation via entanglement routing [1,2,3]. The motivation for integrating quantum communication into 7G stems from its alignment with the envisioned properties of 7G systems: autonomous orchestration, ultra-low-latency reliability, global coverage, and end-to-end security. As conventional technologies approach theoretical performance ceilings, quantum systems offer new possibilities in overcoming channel capacity limits, secure satellite communications, and next-generation Internet-of-Things (IoT) deployments [4,5,6]. Figure 1 illustrates the general concept of quantum computing in which every domain-specific application interacts with domain-independent services, while, in each domain, sensors and actuators communicate directly with each other.

The emergence of quantum communication is the culmination of more than four decades of advances in quantum information science. The field began with the pioneering concept of quantum conjugate coding [7], which first suggested that quantum mechanical principles could provide information-theoretic security. This idea was transformed into a practical communication paradigm through the BB84 protocol [8], establishing Quantum Key Distribution (QKD) as the first application capable of guaranteeing unconditional security based on the laws of quantum mechanics rather than computational assumptions. It was subsequently demonstrated that Bell inequality violations and quantum entanglement could similarly enable secure communication via the E91 protocol [9], thereby fundamentally linking quantum cryptography to the non-local properties of entangled particles.

As research progressed, the community’s focus shifted from secure point-to-point communication toward scalable quantum networking infrastructures. One of the principal obstacles was the exponential attenuation experienced by photons transmitted through optical channels. This challenge was addressed through the introduction of the quantum repeater [10], which combined entanglement swapping and entanglement purification to enable long-distance quantum communication without violating the no-cloning theorem. This breakthrough transformed quantum communication from isolated quantum links into distributed quantum networks capable of supporting entanglement distribution across multiple intermediate nodes. Building upon these developments, the vision of a global Quantum Internet was articulated [11], where heterogeneous quantum devices interconnected through photonic channels would collectively enable distributed quantum computing, secure communication, networked sensing, and cloud-based quantum services.

Since these foundational milestones, quantum networking research has evolved from investigating individual communication protocols toward developing complete networking architectures. Contemporary research encompasses quantum routing, entanglement resource allocation, quantum software-defined networking, distributed quantum computing, programmable control planes, quantum middleware, digital twins, and AI-assisted orchestration [5,12,13]. Parallel advances in simulation platforms such as NetSquid [14], QuNetSim [15], and SeQUeNCe [16] have enabled realistic full-stack evaluation of quantum protocols, allowing researchers to investigate cross-layer interactions between hardware imperfections, network protocols, and application-level services. Consequently, current research no longer views quantum communication as an isolated cryptographic primitive but rather as the communication substrate supporting future quantum internetworks [17,18].

The foundational literature of quantum communication comprises several landmark surveys that any quantum networking study must be situated against. Wehner, Elkouss, and Hanson [13] provided the field with its most-cited architectural roadmap, identifying six staged capability tiers for a quantum internet—from trusted-node QKD through fully fault-tolerant entanglement distribution—and defining the hardware and protocol milestones required at each tier. The survey of entanglement-assisted networks by Li et al. [12] subsequently systematized enabling technologies, cross-layer dependencies, and open research challenges at the protocol level, covering entanglement generation, purification, routing, and session management. Illiano et al. [17] offered a comprehensive treatment of the quantum internet protocol stack that directly motivates the layer-resolved perspective adopted in this survey, while Huang et al. [19] analyzed the same body of work from the layered protocol perspective, clarifying the functional distinctions among substrate, link, network, and application tiers. The authoritative review of quantum repeater architectures by Azuma et al. [20] established the hardware context—spanning three repeater generations across all major physical platforms—within which the Layer 0 substrate design proposed here operates. The Quantum-Converged OSI stack presented in this survey is motivated by the gaps these works collectively identify: no prior work provides a unified, nine-layer protocol framework that explicitly maps QKD, QEC, PQC, and AI orchestration to their respective layers, defines formal inter-layer telemetry APIs, and introduces a Cognitive Intent Plane as a first-class architectural component.

Despite decades of remarkable progress, the transition from point-to-point quantum communication to scalable quantum internetworking remains an open systems problem. While fundamental protocols such as BB84, E91, teleportation, and quantum repeaters successfully demonstrated secure communication and long-distance entanglement distribution, they were largely developed independently, each targeting specific physical or protocol-level challenges. Integrating these heterogeneous technologies into a unified networking architecture comparable to today’s Internet remains considerably more challenging. As a result, integrating quantum communication into the 7G paradigm is not a matter of incremental enhancement. Instead, it requires a layered redesign that spans hardware (e.g., quantum repeaters, metasurfaces) and software (e.g., entanglement routing, quantum session control). Numerous surveys have recently examined specific aspects of quantum networking, including quantum key distribution, post-quantum cryptography, quantum software-defined networking, quantum machine learning, and AI-assisted orchestration. Although these studies provide valuable insights into individual technologies, most adopt a technology-centric perspective and therefore lack a unified architectural framework capable of explaining how these diverse mechanisms collectively support end-to-end quantum networking. Moreover, relatively few surveys discuss the historical evolution of quantum networking architectures, the progression from protocol-centric research toward cross-layer designs, or the interaction between quantum communication principles and the classical networking abstractions inherited from the OSI model. Consequently, a comprehensive architectural perspective that bridges foundational quantum communication principles with emerging quantum-native networking stacks remains absent.

### Limitations of Classical OSI Stack in Quantum Context

The classical OSI model, a cornerstone of modern network protocol design, was developed to support the linear abstraction of digital communication. However, its assumptions fundamentally contradict the properties of quantum information systems. This section outlines the architectural misalignments and their implications for quantum networking in 7G environments.

*(1) No-Cloning Theorem:* Classical networking relies on the duplicability of data packets, enabling retransmissions, caching, and redundancy. In quantum communication, the *no-cloning theorem* prohibits copying arbitrary quantum states, making classical packet duplication, backup, and loss recovery infeasible [21].

*(2) Decoherence and Fidelity Constraints:* Quantum states are inherently fragile and degrade due to interactions with the environment—a process known as *decoherence*. This challenges session persistence, routing stability, and transport reliability. However, the classical OSI stack lacks inherent mechanisms to handle time-sensitive quantum coherence requirements or to model the lifespan of entanglement within protocol flows [12,22].

*(3) Inadequate Error Correction Models:* Classical error correction assumes bit errors and noise can be corrected via parity checks or redundancy schemes. However, Quantum Error Correction (QEC) involves maintaining superpositions of states and requires entirely new constructs, such as surface codes, logical qubits, and entanglement-assisted recovery, whereas OSI layers lack interfaces or representations for QEC codes [23].

*(4) Absence of Entanglement Awareness:* Entanglement introduces non-local correlations that are not addressable in classical routing or addressing models. In the OSI architecture, layers such as Network and Transport operate under the assumption of addressable, isolated endpoints. Quantum links, by contrast, are shared probabilistic resources and require coordination protocols that span multiple layers to preserve entanglement fidelity and coherence [24,25].

**Figure 2:**
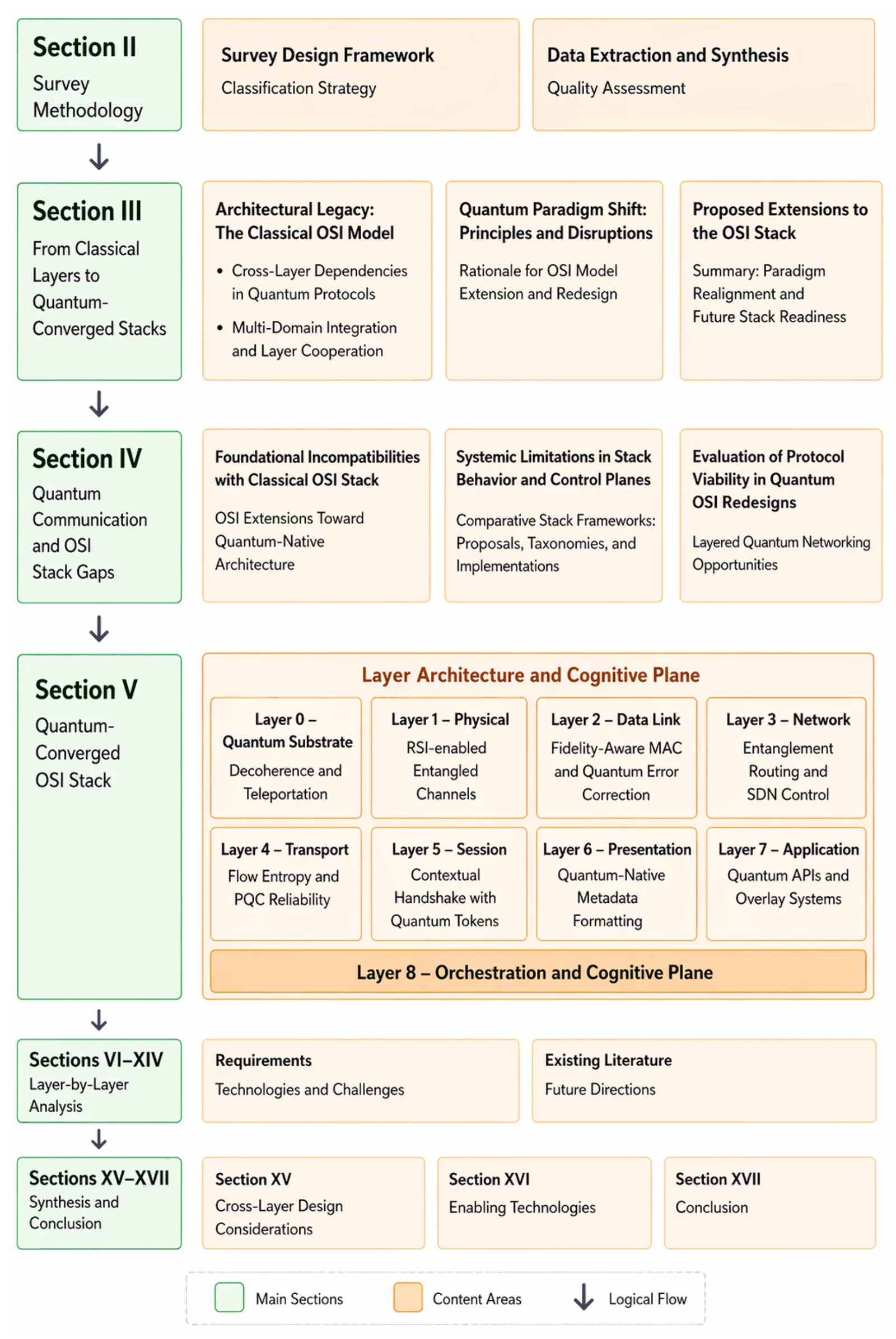
Overview of the paper organization, illustrating the nine-layer Quantum-Converged OSI stack structure and the survey’s coverage of enabling technologies, cross-layer dependencies, simulation tools, and evaluation metrics across Layers 0–8.

*(5) Lack of Cross-Layer Coordination:* The strict layering principle of OSI discourages real-time interaction across layers. In contrast, quantum protocols often require cross-layer feedback (e.g., physical-layer fidelity to influence session control or routing). This necessitates a departure from rigid OSI encapsulation toward a more integrated, dynamically coordinated protocol model [18,26]. The same need for vertical feedback is later formalized in Section 3, where state and performance telemetry flow across the quantum-converged stack instead of remaining trapped within isolated layers. To address these challenges, a quantum-aware redesign of the OSI model is needed to introduce new abstractions, interoperable quantum–classical interfaces, and protocol primitives designed for entanglement-based communication. The summary of relevant abbreviations in this survey is listed in Table 1 and Table 2 and Figure 2 shows the overview of the paper organization.

## 2. Survey Methodology

This section outlines the methodological framework adopted for conducting this layered quantum-converged protocol stack research survey. The methodology is informed by Systematic Literature Review (SLR) practices, combining quantitative and qualitative synthesis from highly curated databases. This survey was conducted in accordance with the Preferred Reporting Items for Systematic Reviews and Meta-Analyses (PRISMA 2020) guidelines. The review process included identification, screening, eligibility assessment, and inclusion of relevant studies. Since this work is a survey-oriented review of the published literature on quantum networking and OSI stack redesign, no formal review protocol registration was conducted.

### 2.1. Survey Design Framework

To ensure methodological rigor, this survey adopts a structured approach inspired by systematic literature review (SLR) frameworks, notably following four principles—replicability, thematic breadth, bias mitigation, and traceability [27]. Drawing on PRISMA guidelines, our process included identification, screening, eligibility assessment, and inclusion. The literature from January 2018 to March 2025 was sourced via advanced keyword searches (e.g., “quantum OSI stack,” “entanglement routing,” “AI in quantum networks”) across IEEE Xplore, SpringerLink, ScienceDirect, ACM DL, and arXiv. Inclusion required peer-reviewed studies that addressed at least one OSI layer in quantum or hybrid systems and made empirical, theoretical, or architectural contributions. Non-peer-reviewed sources and low-depth perspectives were excluded to ensure academic rigor and relevance [19,28]. An initial search yielded 428 records collected from IEEE Xplore, SpringerLink, ScienceDirect, ACM Digital Library, and arXiv using predefined keyword combinations related to quantum networking and OSI architectures. After duplicate removal and preliminary filtering, 372 records remained for title and abstract screening. Following eligibility assessment based on relevance, technical depth, and contribution to quantum-enabled networking architectures, 167 studies were selected for detailed qualitative synthesis and inclusion in this survey.

To ensure reproducibility, we provide the full Boolean search strings employed across all five databases. The primary search string applied to IEEE Xplore, SpringerLink, ScienceDirect, ACM Digital Library, and arXiv was: *(“quantum network” OR “quantum communication”) AND (“OSI” OR “protocol stack” OR “network architecture”) AND (“entanglement” OR “QKD” OR “quantum error correction” OR “decoherence”)*. Secondary search strings targeting specific sub-topics included: *(“quantum internet” OR “quantum repeater”) AND (“routing” OR “fidelity” OR “coherence”) (“post-quantum cryptography” OR “PQC”) AND (“network layer” OR “protocol design”) (“AI” OR “machine learning” OR “LLM”) AND (“quantum network” OR “quantum orchestration”)*. All searches were conducted between January 2018 and March 2025, with filters applied for English-language publications only. The same strings were applied consistently across all databases to minimize selection bias.The literature screening followed a four-stage PRISMA 2020 workflow, with quantitative statistics reported at each stage as follows:

**Stage 1—Identification:** An initial search across five databases yielded n=428 records (IEEE Xplore: 172, SpringerLink: 104, ScienceDirect: 92, ACM Digital Library: 38, arXiv: 22). Duplicate records were identified and removed (n=56), yielding n=372 unique records for screening.
**Stage 2—Title and Abstract Screening:** All 372 records were independently screened by two authors against the predefined inclusion criteria. Records were excluded if they: (i) were unrelated to quantum networking or OSI-stack design (n=98); (ii) addressed only classical network protocols without quantum extensions (n=27); or (iii) were non-English publications (n=15). A total of n=140 records were excluded at this stage, leaving n=232 for full-text assessment. Inter-rater agreement was measured using Cohen’s κ=0.81, indicating strong agreement.
**Stage 3—Full-Text Eligibility Assessment:** Full texts of n=232 records were assessed for eligibility. Records were excluded for the following reasons: not relevant to quantum networking or OSI stack (n=28); insufficient technical depth or usefulness (n=17); not original research, review, or tutorial (n=11); non-English publications identified at full-text stage (n=5); insufficient data or clarity (n=4). A total of n=65 records were excluded, yielding n=167 studies for inclusion.
**Stage 4—Inclusion:** The final corpus of n=167 studies was included in the qualitative synthesis. The overall inclusion rate was 39% of the initial identified records.

Our primary inclusion criterion favored peer-reviewed publications. However, a carefully selected subset of arXiv preprints (n=22 at the identification stage) was considered under the following strict conditions: (i) the preprint had received substantial citation attention within the quantum networking community; (ii) it addressed a topic for which no equivalent peer-reviewed treatment was available within the review window; and (iii) it had been made available by identifiable research groups with established publication records in the field. Each arXiv source retained in the final corpus (n=9 of the 167 included studies) is explicitly flagged in the reference list with the notation [Preprint] to ensure transparency. We have revised the methodology statement to accurately reflect this nuanced inclusion policy, replacing the prior categorical exclusion language with the conditional criteria described above.

### 2.2. Classification Strategy

**Figure 3:**
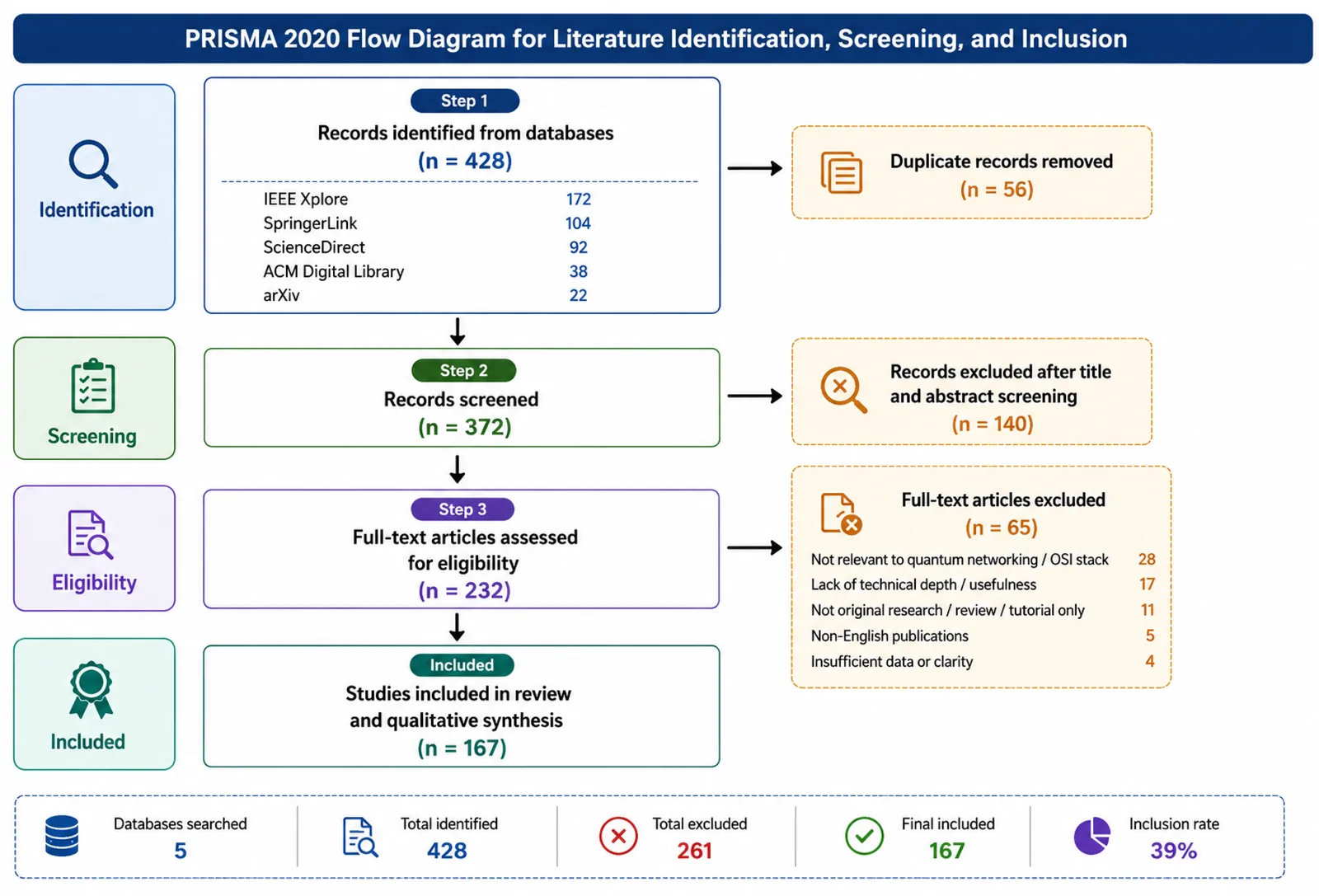
PRISMA 2020 flow diagram illustrating the literature identification, screening, eligibility assessment, and inclusion process adopted in this survey. Stage 1 identified n=428 records across five databases; after removing 56 duplicates, n=372 remained. Stage 2 excluded 140 records leaving n=232; inter-rater agreement was Cohen’s κ=0.81. Stage 3 excluded 65 records yielding n=167. Stage 4 included the final corpus of n=167 studies (39% of identified records) in the qualitative synthesis. Arrows indicate the direction of record flow between the four PRISMA stages; the boxes to the right of each stage list the records excluded at that stage. Colours are used only to distinguish the stages and carry no additional meaning.

Given the multidisciplinary scope of quantum networking, we adopt a structured three-axis classification to organize the surveyed literature. The first axis, Layer Mapping, aligns each contribution with one or more OSI layers—including our proposed extensions: Layer 0 (Quantum Substrate) and Layer 8 (Cognitive Intent Plane)—to facilitate stack-wise analysis and identify research density or gaps across the protocol stack. The second axis, Technology Domains, categorizes work by its core enabler, enabling comparison of technology distribution across layers. The third axis, Application Use Cases, maps contributions to practical deployment contexts such as satellite-based QKD, entangled UAV networks, quantum IoT systems, and programmable infrastructure for the quantum internet. Figure 3 summarizes the PRISMA-based study selection and screening workflow used in this survey.

### 2.3. Quality Assessment

We adopted a structured quality assessment framework to ensure rigor and consistency, inspired by systematic review practices in quantum computing and communication. Following [21], each paper was evaluated against five protocol-stack-specific dimensions:

1. *Relevance:* Contribution to quantum networking architecture, protocols, or orchestration.
2. *Layer Specificity:* Explicit focus on one or more OSI layers (including proposed extensions) or cross-layer dependencies.
3. *Modeling/Implementation Clarity:* Preference for works with protocol models, empirical validation, or simulation frameworks (e.g., NetSquid, QuNetSim, QuISP).
4. *Innovation:* Novelty in hybrid-stack design, AI-based control, quantum SDN, or fidelity-aware orchestration.
5. *Rigor:* Methodological soundness judged by peer-review status, citation strength, and technical depth.

To provide structured and transparent quality assessment outputs, each of the 167 included studies was scored against the five assessment dimensions defined in Section 2.3, using a three-point scale: 0 (not addressed), 1 (partially addressed), 2 (fully addressed). Studies with an aggregate score below 6 out of 10 were flagged for supplementary review. Table 3 summarizes the distribution of quality scores across the included corpus. Studies scoring below the threshold (n=14) were retained only where they provided a unique architectural perspectives unavailable in higher-scoring works, and their limitations are explicitly noted where cited.

Table 4 positions the present survey against representative quantum-networking surveys across the six evaluation dimensions introduced above.

## 3. Quantum Communication and OSI Stack Gaps

**Figure 4:**
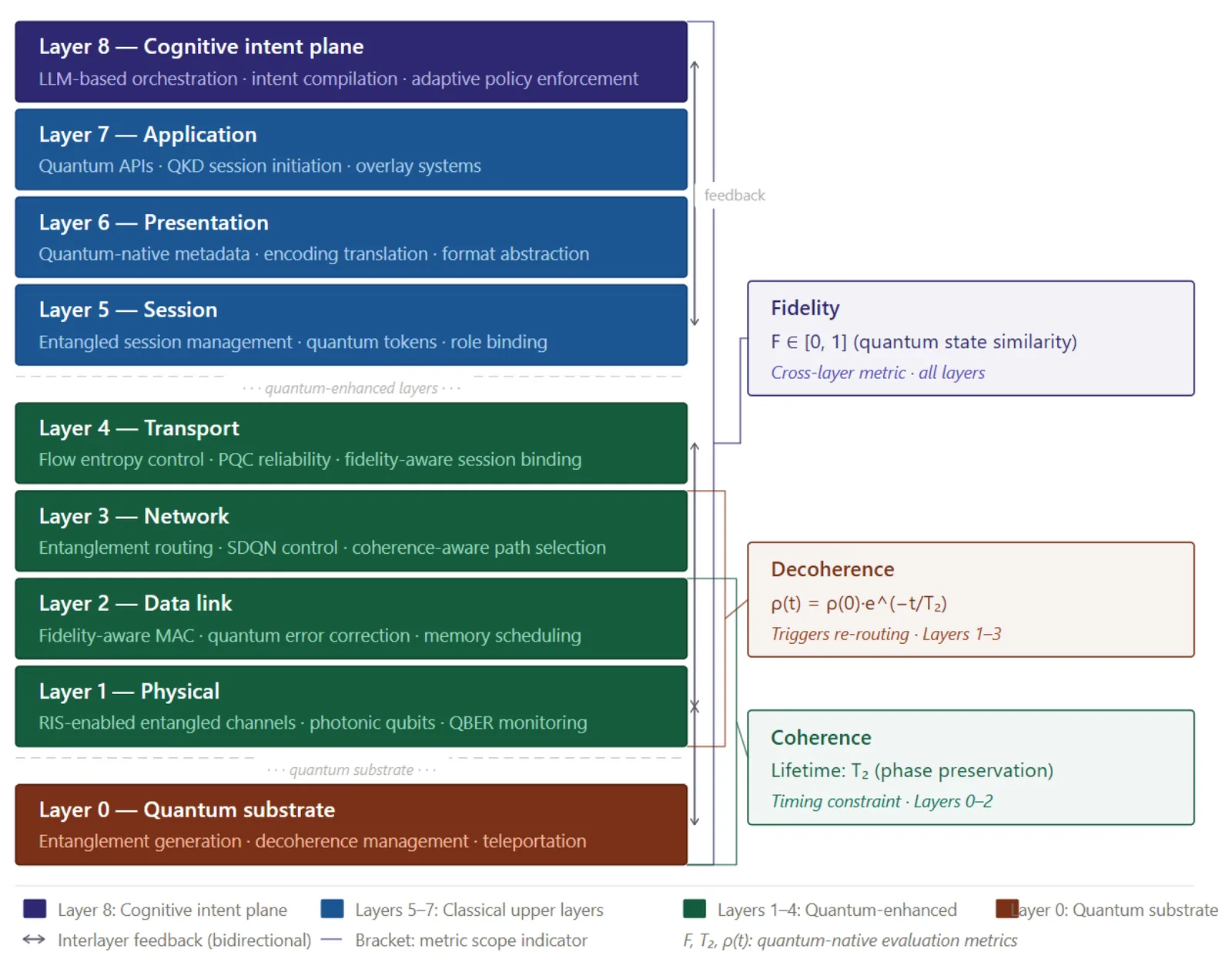
Feedback-coupled quantum-converged OSI reference model illustrating the three primary cross-layer control signals. Decoherence (annotated at Layers 1–3) refers to environmentally induced loss of quantum phase coherence modeled as exponential decay with characteristic time $T_{2}$, manifesting as increased QBER and triggering rerouting at Layer 3 or session renegotiation at Layer 5. Coherence (annotated at Layers 0–2) refers to preservation of superposition states, with $T_{2}$ defining the maximum permissible latency for multi-hop entanglement swapping. Fidelity (annotated across all layers) serves as the primary cross-layer quality metric propagated via telemetry APIs; a drop below $F_{\min}$ triggers encoding downgrades at Layer 6, session pausing at Layer 5, or entanglement path switching at Layer 3, as formalized in Section 13.

Quantum communication introduces phenomena such as entanglement, coherence decay, and the no-cloning principle that fundamentally violate classical OSI assumptions. The OSI stack’s modularity and determinism conflict with quantum properties that are probabilistic, non-replicable, and time-sensitive. For example, the no-cloning theorem precludes retransmission protocols at Layers 2 and 4 [34], while coherence loss mandates temporal coupling across layers [35]. Classical error correction fails under quantum noise types like phase flip and depolarization [36]. These issues demand architectural realignment: authors in [17,24] advocate adding new layers for semantic control and cross-stack orchestration. As quantum networks scale—from satellite QKD to integrated testbeds [37]—this section explores OSI-stack limitations and introduces Layer 0 (Quantum Substrate) and Layer 8 (Cognitive Intent Plane) as foundational extensions. The resulting stack-wide control pattern is summarized in Figure 4, which frames these limitations as feedback paths rather than isolated layer faults.

Figure 4 illustrates the proposed feedback-coupled quantum-converged OSI reference model. The three annotations carry the following precise interpretations at the protocol level:

**Decoherence** (annotated at Layers 1–3) refers to the environmentally induced loss of quantum phase coherence in transmitted qubits, modeled as an exponential decay with a characteristic time $T_{2}$. At the protocol level, this manifests as an increase in QBER and a reduction in entanglement fidelity, triggering rerouting or session renegotiation at Layers 3 and 5 respectively.
**Coherence** (annotated at Layers 0–2) refers to the preservation of superposition states across memory and transmission operations. Protocol implications include strict timing constraints on entanglement swapping and memory scheduling, as  coherence windows define the maximum permissible latency for multi-hop operations.
**Fidelity** (annotated across all layers) serves as the primary cross-layer quality metric, propagated upward via telemetry APIs. A  fidelity drop below a predefined threshold $F_{\min}$ at any layer triggers adaptive responses at Layer 8, including encoding downgrades (Layer 6), session pausing (Layer 5), or  entanglement path switching (Layer 3).

### 3.1. Foundational Incompatibilities with Classical OSI Stack

Quantum networking fundamentally challenges classical OSI assumptions due to physical constraints such as the no-cloning theorem, coherence fragility, and nonclassical noise models. These limitations permeate all layers and require a rethink of systemic protocols.

*No-Cloning Constraint.* The no-cloning theorem prohibits duplicating unknown quantum states, undermining packet retransmission and duplication mechanisms central to Layers 2 and 4. The authors in [36] show that ARQ and TCP-style recovery are invalid in quantum contexts, where state collapse on measurement precludes retransmission.

*Temporal Coherence Sensitivity.* Qubit decoherence imposes strict timing requirements, necessitating cross-layer synchronization. The authors in [23] highlighted that coherence preservation over satellite–ground links demands real-time coordination, particularly during channel switching or entanglement relaying.

*Quantum Error Models.* Classical error correction fails in the face of quantum-specific noise—depolarization, phase flips, amplitude damping. The authors in [38] emphasize the need for surface, Shor, or Steane codes as integrated, stack-aware modules, anchored in real-time physical-layer feedback. The surface code family referenced throughout this survey is among the most practically important of these constructs, and its fault-tolerant threshold properties under realistic noise models have been established in [39]. Modular decoding approaches that reduce the syndrome-extraction overhead to make QEC compatible with the memory-scheduling constraints of quantum link layers have been proposed in [40]. A critical and under-appreciated architectural point is that QEC codes consume coherence time during each syndrome-extraction round; the memory aging model at Layer 2 must therefore be parameterized by the QEC cycle duration, creating a cross-layer dependency between the error-correction subsystem and the MAC scheduler that has no analogue in classical networks.

*Stateful, Entanglement-Aware Routing.* Classical stateless routing fails in quantum contexts, where entanglement fidelity and swapping strategies must inform route selection. The authors in [24] argue for routing protocols that are inherently stateful and context-aware, attuned to coherence and fidelity dynamics.

### 3.2. Limitations in Stack Behavior and Control Planes

Beyond structural mismatches, quantum communication disrupts OSI operational assumptions, particularly regarding control logic and session orchestration. Classical networks rely on deterministic protocols, stateless feedback, and modular isolation—assumptions incompatible with quantum systems’ probabilistic behavior and their sensitivity to coherence.

*Deterministic Control Limitations:* Traditional protocols (e.g., OSPF, BGP, TCP) assume predictable channel states. Yet quantum networks face measurement collapse and stochastic entanglement success. The authors in [41] and the authors in [42] argue for control planes with hybrid, probabilistic logic capable of adapting to non-deterministic transmission dynamics.

*Lack of Fidelity Feedback:* Classical metrics like RTT and link state are insufficient in quantum contexts, which require fidelity, QBER, and coherence telemetry. The authors in [17] highlight the need for cross-layer fidelity-aware feedback to support real-time path adaptation and entanglement orchestration—features absent in legacy stacks.

*Semantic Unawareness at Layer 7:* Classical application layers are syntactic, decoupled from physical-layer semantics. Quantum applications, however, rely on context-sensitive coherence, fidelity budgets, and quantum state alignment. The authors in [43] call for semantically adaptive layers capable of coordinating teleportation tasks, QKD setup, and fidelity-aware sessions.

### 3.3. OSI Extensions Toward Quantum-Native Architectures

As quantum communication exposes the OSI model’s structural inadequacies, researchers propose architectural augmentations to support coherence-sensitive, probabilistic operations. Chief among these are the addition of Layer 0 and Layer 8—framing the stack with substrate-level quantum control and semantic-intent orchestration, respectively—alongside middleware frameworks for quantum–classical integration. *Layer 0* formalizes hardware-near abstractions to manage entanglement fidelity, coherence time, and teleportation channels. The authors in [44] define it as a programmable substrate interfacing with quantum devices and exposing telemetry (e.g., qubit freshness, error rates) to upper layers for real-time adaptation. *Layer 8*, the Cognitive Intent Plane, translates user goals into protocol flows using AI reasoning and semantic orchestration. It enables policy-driven updates based on constraints like fidelity margins, privacy thresholds, and entanglement availability [45], forming a closed semantic feedback loop with lower layers. To bridge hybrid deployments, middleware sidecars have emerged as translation agents between quantum and classical stacks. The authors in [24] propose sidecar designs that expose standardized APIs for SDN, PQC, and blockchain integration, supporting stackless emulation, identity handling, and secure control over entangled and classical flows.

### 3.4. Comparative Stack Frameworks

As the OSI model proves insufficient for quantum networking, various stack frameworks have emerged to bridge the gaps between classical and quantum architectures. These range from minimally adapted classical models to quantum-native stacks with novel APIs, control logic, and semantic layers. This section synthesizes prominent contributions and offers a taxonomy of modular, hybrid, and standards-driven approaches to quantum network design.

*Modular Stack Models.* Pirker and Dür proposed an early modular quantum stack with composable layers for fidelity tracking, teleportation, and entanglement routing [46]. Building on this, the authors in [17] introduced real-time telemetry and cross-layer entropy feedback to enable decentralized, fidelity-aware routing decisions.

*Hybrid Quantum-Classical Stack Designs.* The authors in [24] presented a hybrid OSI-compliant architecture embedding quantum control in vertical slices while retaining classical control paths. Their model defines APIs for session coordination, resource mapping, and legacy compatibility.

*Emerging Standards: QIR, QCoDeS, and SDQN.* The authors in [47] advocate for Software-Defined Quantum Networking (SDQN), combining centralized control, intent-aware orchestration, and programmable entanglement interfaces. Toolchains like QIR and QCoDeS are highlighted as critical enablers for quantum–classical integration.

The proposed Quantum-Converged OSI stack advances beyond existing frameworks along three principal dimensions. First, where Pirker and Dür’s modular quantum network model [46] introduces composable entanglement layers but does not address orchestration or cognitive control, and where Caleffi et al.’s SDQN framework [48] provides a programmable control plane but is limited to Layers 1–3, the proposed model extends the stack in both directions simultaneously, downward to Layer 0 for substrate-level quantum state management and upward to Layer 8 for LLM- and QML-assisted intent compilation and closed-loop reconfiguration. Second, unlike Dahlberg et al.’s link-layer stack [49], which formalizes Layer 2 entanglement generation protocols but treats higher layers informally, the proposed architecture provides explicit inter-layer interface primitives and telemetry APIs for all nine layers, enabling stack-wide fidelity propagation and coherence-aware orchestration. Third, in contrast to QKD network architectures such as SECOQC and OpenQKD, which operate primarily at the key-distribution level without a unified protocol stack, the proposed model integrates QKD, QEC, PQC, and SDQN as layer-specific components within a coherent architectural framework, explicitly mapping each technology to its functional layer and defining the cross-layer dependencies that govern its interaction with adjacent protocol functions.

Two additional benchmarks that any serious quantum networking architecture must be positioned against are the Dahlberg et al. link-layer protocol [49] and the experimental demonstration of entanglement delivery using a quantum network stack [35]. Dahlberg et al. formalized the first rigorous link-layer protocol for quantum networks at SIGCOMM 2019, defining a suite of service primitives—*create entanglement*, *measure*, and *connect*—that map naturally onto the Layer 2 MAC and memory-management functions described in this survey. Pompili et al. subsequently implemented those link-layer primitives end-to-end across three NV-center nodes, demonstrating that entanglement can be delivered as a reliable network service rather than a point-to-point physical phenomenon. Together, these two works define the link-layer and experimental baseline from which the nine-layer stack in this survey extends: they treat Layers 0 and 2 rigorously, but leave Layers 3–8 as open design problems that the Quantum-Converged OSI stack directly addresses. The broad quantum networking literature has been comprehensively surveyed from a layered protocol perspective in [17,19], both of which confirm that no prior framework provides unified layer definitions for all nine tiers—from substrate entanglement management through AI-assisted cognitive orchestration—with formal inter-layer interface primitives at each boundary.

To contextualize our unified OSI redesign, Table 5 summarizes leading projects and protocols across key quantum networking challenges.

*Emerging Standards: QIR, QCoDeS, and SDQN* Brito and collaborators have outlined a blueprint for scalable quantum network deployments by leveraging SDN paradigms in quantum systems. Their model includes centralized control loops, intent-aware service orchestration, and programmable interfaces for entanglement allocation. They also examine emerging toolchains such as Microsoft’s QIR and QCoDeS as essential building blocks for hardware–software integration across the quantum stack [47].

### 3.5. Protocol Viability in Quantum OSI Redesigns

Designing viable quantum protocol stacks demands both architectural innovation and empirical validation using simulation environments and quantum-specific performance metrics. Classical testing methods fail to capture coherence-aware behaviors, necessitating tools like QuNetSim for lightweight entanglement routing prototyping [15], NetSquid for high-fidelity noise modeling and teleportation orchestration [14], SimulaQron for distributed quantum execution via qubit sockets [51], and QuISP for programmable fidelity-based stack scheduling [52]. These simulators span various abstraction levels and support critical evaluation tasks. Performance assessment hinges on quantum-native metrics: fidelity as the baseline for state similarity [53], fidelity drift as a temporal entropy effect [54], entropy rate as a decoherence leakage index [55], probabilistic throughput optimized via reinforcement learning [56], and coherence cost tied to entropy dissipation in spin-based systems [57]. However, protocol traceability remains a challenge due to quantum measurement collapse. Tools like NetSquid and SimulaQron allow limited temporal tracking (e.g., entanglement lifetime), but standardized, non-invasive debugging aids such as coherence monitors and fidelity beacons are urgently needed [19] to ensure stack observability across dynamic, multi-hop architectures.

## 4. Quantum-Converged OSI Stack

**Figure 5:**
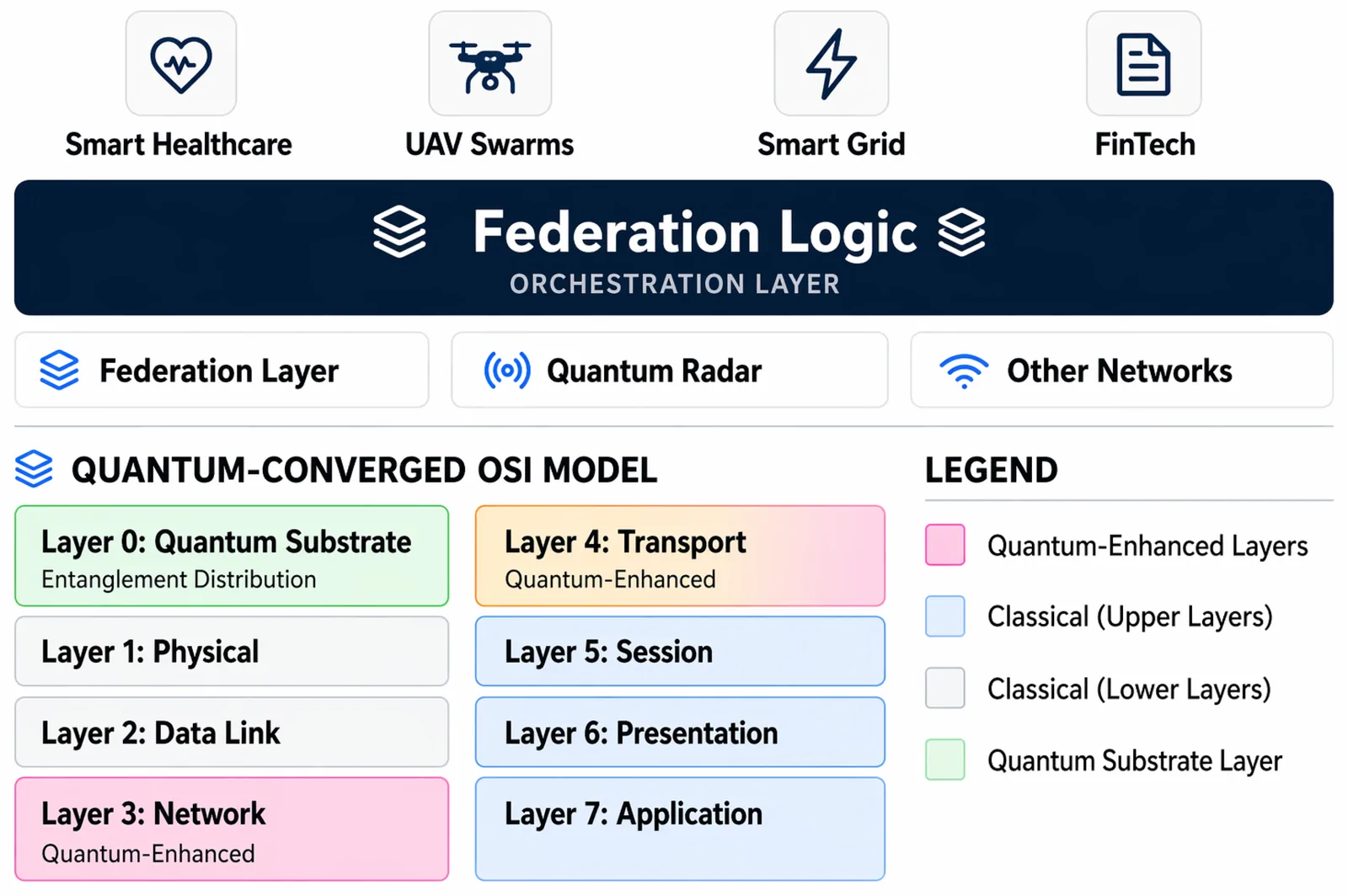
Federated quantum applications supported by the proposed Quantum-Converged OSI stack. Application domains such as Smart Healthcare, UAV Swarms, Smart Grids, and FinTech access shared quantum-network services through a federation layer that coordinates entanglement resources, session management, security policies, and cross-domain orchestration. Colours are used only to separate the vertical application domains from the horizontal shared-infrastructure layers and carry no additional quantitative meaning; the shading of the Layer 4 band marks the transport functions that are common to all domains.

Quantum networking demands a restructured OSI-like stack that integrates quantum-specific constraints such as decoherence, fidelity, and probabilistic entanglement into each protocol layer. This work proposes a nine-layer model spanning Layer 0 (Quantum Substrate) to Layer 8 (Cognitive Intent Plane), encompassing both physical infrastructure and AI-driven orchestration. Layer 0 abstracts physical qubit channels, entanglement interfaces, and memory coupling [58,59], while Layer 8 leverages LLMs for semantic intent translation and adaptive stack control [17,60]. Intermediate layers are revisited under quantum constraints: Layer 3 supports coherence-aware entanglement routing [61], and Layer 5 enables session control via quantum metadata and identity tokens [62]. Cross-layer feedback—once discouraged in classical stacks—is now essential for real-time orchestration over dynamic, entropy-sensitive paths [63,64]. The following Section 5, Section 6, Section 7, Section 8, Section 9, Section 10, Section 11, Section 12 and Section 13 detail each layer’s quantum roles, technologies, and architectural trends. Figure 5 illustrates how diverse quantum domains interface via the federation logic built on top of the Quantum-Converged OSI stack’s upper layers.

To ensure scientific rigor, we formally define the core quantum-native metrics used throughout this survey. Entanglement fidelity between a transmitted state ρ and the ideal target state |ψ〉 is defined as:

(1) F(ρ,|ψ〉)=〈ψ|ρ|ψ〉∈[0,1]

where F=1 denotes perfect state preservation and F=0 denotes complete state collapse. Coherence lifetime $T_{2}$ characterizes the timescale over which a qubit maintains phase coherence, governed by

(2) $$\rho \left(t\right)=\rho \left(0\right)\cdot e^{-t/T_{2}}$$

The Quantum Bit Error Rate (QBER) is defined as the ratio of erroneous bits to the total number of bits received:

(3) $$\mathrm{QBER}=\frac{N_{\mathrm{error}}}{N_{\mathrm{total}}}$$

with acceptable thresholds typically below 11% for BB84-based QKD protocols. As QBER increases under decoherence and channel loss, the entanglement fidelity *F* decreases correspondingly, reducing the probability of successful key generation or teleportation; once QBER exceeds the protocol threshold, the session is no longer viable and triggers rerouting or renegotiation at the network and session layers. Entropy throughput, representing the rate of quantum information transfer under decoherence constraints, is expressed as

(4) $$S_{\mathrm{throughput}}=-\sum_{i}\lambda_{i}\log_{2}\lambda_{i}\cdot R$$

where $\lambda_{i}$ are the eigenvalues of the density matrix ρ and *R* is the raw transmission rate. Routing entropy is defined as

(5) $$S_{r}=-\sum_{i}p_{i}\log p_{i}$$

where $p_{i}$ is the probability of selecting entanglement path *i* under the current routing policy; higher values indicate more balanced load distribution and this metric is consumed by Layer 8 for proactive load-balancing decisions. Teleportation delay $D_{\mathrm{tp}}$ is defined as the end-to-end latency from teleportation request initiation to classical correction completion, encompassing Bell-state measurement time, classical channel propagation delay, and correction gate execution time; it is the primary application-level latency metric exported by Layer 7 and enforced via SLA contracts at Layer 8. These metrics collectively form the evaluation framework applied across Layers 0–8 in the subsequent sections.

Table 6 defines the function of each layer of the proposed stack.

To illustrate how these metrics operate in practice, consider a three-hop entanglement path $A\to R_{1}\to R_{2}\to B$ over fiber links with $T_{2}=50\;\mathrm{ms}$ memory coherence at each repeater node and a per-link raw QBER of 7%. Entanglement fidelity after two swapping operations degrades from the initial $F_{0}=0.98$ to approximately $F=F_{0}^{3}\approx 0.94$, remaining above the threshold $F_{\min}=0.90$ required for BB84 key generation. If decoherence at $R_{1}$ increases the per-link QBER to 9%, the aggregate QBER approaches the 11% BB84 threshold; Layer 8 detects this via the telemetry API and issues a reroute(path_id) primitive to Layer 3, selecting an alternative path with lower per-link QBER. Entropy throughput on the original path, computed as $S_{\mathrm{throughput}}=-\sum_{i}\lambda_{i}\log_{2}\lambda_{i}\cdot R$ with $R=10^{4}$ Bell pairs per second, yields a usable rate of approximately 6500 pairs per second after accounting for decoherence-induced state mixing. Routing entropy $S_{r}=-\sum_{i}p_{i}\log p_{i}$ across three candidate paths serves as the load-balancing signal consumed by Layer 8. Teleportation delay $D_{\mathrm{tp}}$ on the three-hop path, encompassing BSM time, two classical channel propagation delays, and  correction gate execution, sets the SLA latency bound enforced at Layer 8. This example demonstrates that the five metrics are not independent: a QBER increase propagates to a fidelity decrease, which falls below $F_{\min}$, triggering a routing-entropy-guided path switch that resets $D_{\mathrm{tp}}$ and restores $S_{\mathrm{throughput}}$ above the application’s SLA threshold.

Table 7 lists the principal elements of the quantum stack together with representative technologies and concepts associated with each.

Table 8 specifies the upward and downward interface primitives exchanged at each adjacent layer boundary in the proposed stack, clarifying the functional distinction between layers that share related concerns such as Layers 0–1 (state existence vs. state propagation) and Layers 2–3 (per-link fidelity measurement vs. fidelity-aware path selection).

To improve readability, all tables in this survey adopt a unified formatting convention: abbreviations follow the definitions in Table 1 and Table 2, symbols are consistent with the metric definitions in Section 4, and  multi-row entries are avoided where a single concise descriptor suffices.

## 5. Layer 0: Quantum Substrate

**Figure 6:**
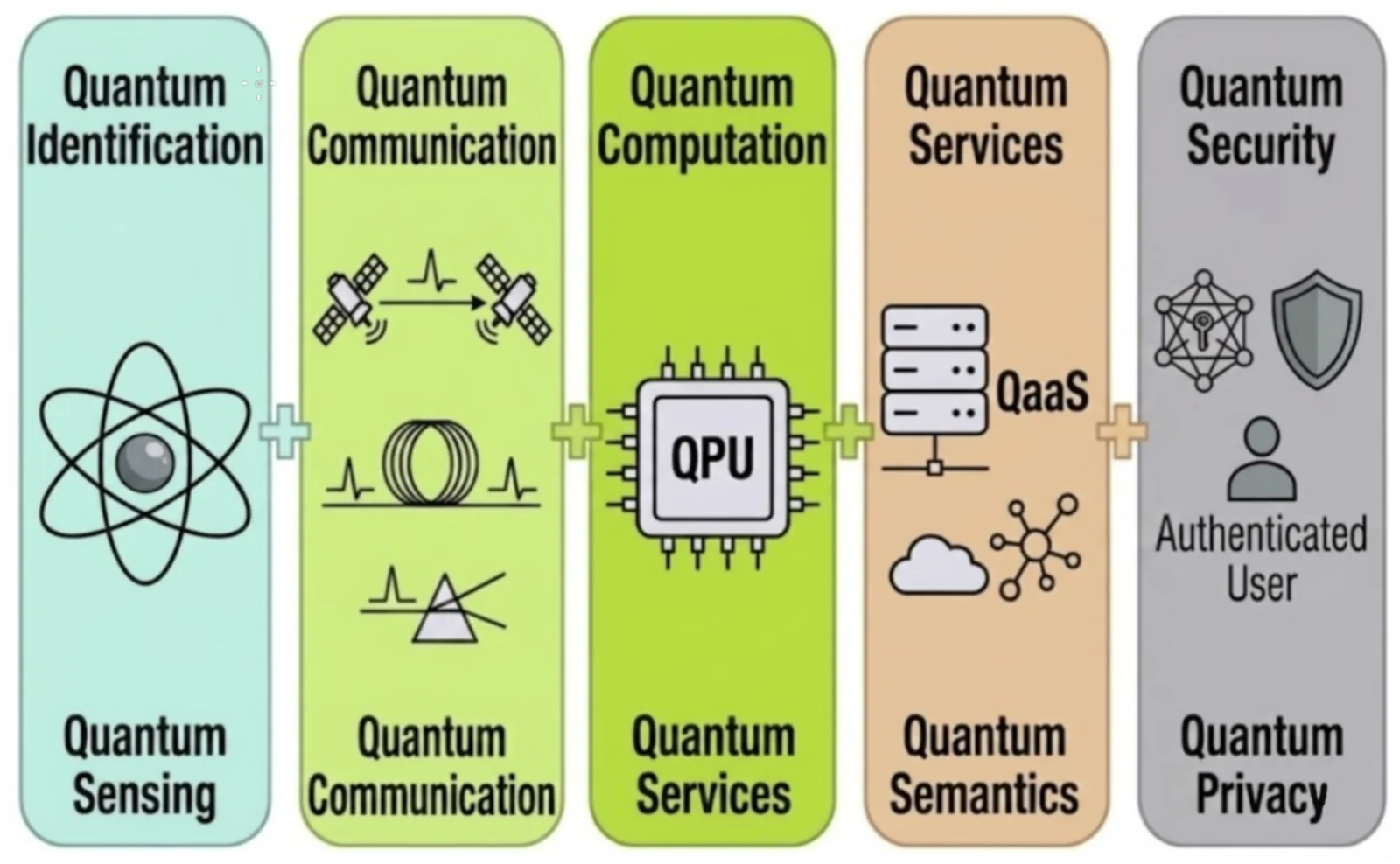
Representative implementations of the Layer 0 quantum substrate. Each technology provides a distinct physical realization of qubits and entanglement resources, with different constraints on coherence time, error rates, and scalability. Trapped ions offer coherence times exceeding 1 s with high fidelity; NV centers provide metropolitan-scale entanglement with medium-high fidelity; SPDC photonics enable long-distance fiber and satellite links with high scalability.

The Quantum Substrate (Layer 0) forms the foundation of the Quantum-Converged OSI architecture, interfacing directly with quantum hardware and entanglement distribution systems. Unlike classical stacks, it must address the fragile, non-clonable nature of quantum states [11,13]. This layer supports entanglement generation, stabilization, and teleportation processes critical for QKD and memory synchronization [74,75]. Key metrics include fidelity, coherence time, and teleportation success, shaped by hardware noise and environmental factors [76]. Implementations span trapped ions [77], NV centers [78], and photonic SPDC sources [79,80], enabling scalable entangled links across diverse topologies. Representative platforms are illustrated in Figure 6. Several simulation and emulation platforms, such as NetSquid [14], have been developed to model Layer 0 behavior under realistic noise, latency, and fidelity conditions. Protocols such as BB84 and E91 serve as practical demonstrations of quantum communication’s physical-layer dynamics, though their scalability remains tightly coupled with advances in substrate-level entanglement and repeater networks [8,9,10]. As quantum networks scale, Layer 0 is projected to evolve from monolithic links to modular, programmable substrates capable of autonomously negotiating entangled routes and self-healing decoherence-induced degradation. Integration with upper-layer orchestration, such as Layer 8’s cognitive intent plane, is expected to enable AI-enhanced management of fidelity, coherence, and entropy constraints [28,46]. The following subsections delve deeper into the requirements, existing literature, enabling technologies, and future research directions for this crucial foundational layer.

*A. Requirements:* Layer 0, the Quantum Substrate, underpins the entire quantum networking stack by handling entanglement generation, quantum state preservation, and transmission through a physical medium. Unlike the classical physical layer, the substrate must maintain fragile quantum states and mediate the probabilistic behavior of entangled communication channels. The design of this layer is constrained by physical phenomena such as decoherence, noise, and the no-cloning theorem [11,13]. Despite these physical constraints, a primary requirement is the ability to distribute entangled qubits across nodes, supporting entanglement swapping, purification, and teleportation [10,74,75]. Moreover, entanglement generation must be high-fidelity and compatible with multiple physical platforms such as trapped ions [77], NV centers in diamond [78], superconducting qubits, and photonic systems using SPDC sources [79,80]. Additionally, substrate protocols must synchronize with quantum memories to allow for storage and scheduling of quantum states.

Another key requirement is ensuring resilience against environmental perturbations. Quantum coherence must be preserved over communication channels such as optical fiber, free-space links, or satellite–ground optical paths [80]. Moreover, this layer must accommodate real-time monitoring and feedback of quantum fidelity and entropy to enable higher-layer orchestration and routing protocols [28,46]. Layer 0 must also facilitate interoperation with quantum-classical hybrid devices through well-defined APIs, allowing stack-wide communication of coherence metrics, memory availability, and entanglement states [14]. These properties form the basis for SDQN, which adapts fidelity-aware routing and control based on substrate-level statistics [28]. Lastly, scalability and modularity are essential to support large-scale entangled topologies. The substrate must efficiently support quantum repeater chaining, node multiplexing, and entanglement distribution protocols over heterogeneous hardware [10,13].

*B. Existing Literature:* The foundational concept of Layer 0 as a quantum substrate derives from the broader vision of a global quantum internet, where physical entanglement channels serve as the communication backbone [11,13]. Early theoretical models such as quantum teleportation [81], quantum repeaters [10], and entanglement purification [82] established the necessity for a dedicated physical layer that manages fragile quantum states with strict coherence and fidelity requirements. In addition, numerous experimental platforms have explored the physical instantiation of this layer. Moreover, trapped ion systems have demonstrated robust quantum coherence and modular entanglement [77]. In contrast, NV centers in diamond offer solid-state alternatives with long coherence times and high-fidelity memory capabilities [78]. However, photonic systems, particularly those using SPDC, have enabled entanglement distribution over terrestrial and satellite links [79,80], highlighting the potential for long-distance, Layer-0 communication. Architectural abstractions have emerged to formalize the role of Layer 0 within network stacks. Pirker and Dür proposed a modular quantum network framework where entanglement links are structured into stackable resource layers, implicitly corresponding to the quantum substrate [83].

Simulation tools have become instrumental in modeling the behavior and performance of the quantum substrate. NetSquid enables discrete-event simulation of entanglement distribution, including physical-layer metrics like memory lifetimes and optical losses [14]. QuNetSim and QuISP offer modular APIs and hardware emulation layers to replicate Layer 0 behavior in hybrid classical–quantum simulations. These platforms allow researchers to test protocols such as BB84 and E91 under realistic physical conditions, providing critical insights into the operational characteristics of Layer 0 components. In addition, a growing body of literature also investigates the coupling between Layer 0 and emergent quantum protocols such as QKD, quantum sensing, and distributed quantum computation. These studies emphasize the importance of accurate decoherence modeling and the need for fidelity-aware protocol stacks [75].

*C. Technologies and Challenges:* The technological foundation of Layer 0 comprises a diverse array of quantum hardware platforms and communication primitives, each tailored to different trade-offs in coherence, fidelity, and distance. Prominent physical qubit technologies include trapped ions [77], NV centers in diamond [78], superconducting transmon qubits [84], and photonic systems using single-photon sources and entangled photon pairs via SPDC [79,80]. Entanglement generation is typically achieved through SPDC, quantum dot emission, or ion–photon entanglement techniques, while entanglement swapping and purification require high-fidelity Bell-state measurement (BSM) circuits and quantum memories [10,75]. These memories—often implemented using atomic ensembles, cryogenic solid-state systems, or spin–photon interfaces—must maintain coherence for durations compatible with round-trip communication delays in multi-hop networks. Quantum repeaters represent a cornerstone technology for extending entanglement across long distances. However, their implementation remains constrained by high-loss channels, gate infidelity, and memory decoherence [74,82]. Most repeater architectures also suffer from low throughput and significant latency due to the probabilistic nature of entanglement generation and the need for heralding and feedback.

Another critical challenge is managing quantum decoherence—loss of information in a quantum system due to environmental interactions. While NV centers and trapped ions provide relatively long coherence times under cryogenic or ultra-high vacuum conditions, they are challenging to integrate into large-scale, mobile, or power-efficient networks [77,78]. On the other hand, photonic systems offer room-temperature operation and higher scalability but face issues like photon loss, indistinguishability, and mode mismatch [79,85]. Additionally, the Layer 0 substrate must also incorporate low-loss optical fibers, free-space optical systems, and satellite–ground links to distribute entanglement across diverse geographic and topological domains [80,86].

The critical role of these substrate-level technologies was validated through a sequence of landmark experimental achievements. Bernien et al. demonstrated heralded entanglement between two NV-center spin qubits separated by three metres via photon-mediated heralding [87], establishing the remote entanglement generation primitive that all multi-node quantum networks require. That NV-center platform was subsequently extended to the three-node entanglement-stack demonstration reported in [35], in which two-hop entanglement swapping across multiple nodes was performed using a full link-layer protocol, providing the first experimental validation of a quantum network stack as an architectural concept rather than a theoretical proposal. At the satellite scale, the Micius mission demonstrated entanglement distribution over 1200 km of free space [80] and satellite-to-ground QKD over intercontinental distances [86], validating the free-space optical Layer 0/Layer 1 path. Taken together, these milestones show that every functional primitive abstracted by Layer 0 in Table 9—entanglement generation, memory-assisted swapping, and long-range distribution—has been demonstrated experimentally on at least one platform, shifting the engineering challenge from proof-of-principle to scalability, integration, and cross-layer protocol design.

### 5.1. Multipartite Entangled Resource States for Quantum Networking

Beyond bipartite Bell pairs, scalable quantum networking architectures increasingly rely on multipartite entangled resource states as substrate-level primitives. The importance of multipartite entanglement for quantum networks was established in [88], who proved that three-qubit pure states fall into exactly two inequivalent entanglement classes under local operations and classical communication (LOCC): the GHZ class and the W class. This inequivalence has direct architectural implications for quantum network design, as the two classes offer complementary robustness properties under realistic photon loss and decoherence conditions.

#### 5.1.1. W States: Definition, Properties, and Networking Relevance

The *n*-qubit W state is defined as [88]:

(6) $$|W_{n}\rangle \;=\;\frac{1}{\sqrt{n}}\left(|10\cdots 0\rangle \;+\;|01\cdots 0\rangle \;+\;\cdots \;+\;|00\cdots 1\rangle \right).$$

A defining and practically important property of $|W_{n}\rangle$ is its robustness to qubit loss. Tracing out any single qubit from $|W_{n}\rangle$ yields a mixed state that retains bipartite entanglement among the remaining n−1 qubits [88]. This stands in sharp contrast to GHZ states, which collapse to a fully separable mixture upon the loss of a single qubit. This loss-tolerance property makes W states particularly attractive as resource states for quantum repeater chains and multi-node quantum networks operating under realistic photon loss and memory decoherence.

*Experimental Realizations of W States.*

W states have been realized experimentally across several physical platforms directly relevant to the Layer 0 substrate. The first experimental realization of a three-photon W state using SPDC and linear-optical post-selection was reported in [89], confirming the theoretical entanglement structure predicted in [88]. The scalable preparation of W states among up to eight trapped ions using laser-driven entangling gates, achieving high fidelity across the full register, was demonstrated in [90], establishing trapped-ion platforms as a leading candidate for deterministic multipartite entanglement generation at the quantum substrate level. Heralded entanglement generation between two NV-center qubits separated by three metres using photon-mediated heralding was demonstrated in [87]; the heralding scheme employed is the foundational primitive upon which multipartite entanglement generation across metropolitan-scale fiber links is being built, providing a scalable mechanism for entangling distant matter qubits without direct interaction.

*Implications for the Quantum-Converged OSI Stack.*

- **Layer 0 (Quantum Substrate):** W-state generation is a primary substrate-level function with platform-specific requirements: trapped-ion systems support deterministic generation via laser-driven entangling gates [90], while photonic systems rely on probabilistic SPDC-based generation [89]. Layer 0 must expose generation fidelity and success probability to higher layers via standardized telemetry APIs.
- **Layer 2 (Data Link):** The probabilistic nature of linear-optical W-state generation necessitates fidelity-aware MAC protocols capable of scheduling multiple generation attempts and managing quantum memory lifetimes across failed rounds, with  garbage collection triggered when stored entanglement ages below the fidelity threshold [89].
- **Layer 3 (Network):** Since W states and GHZ states exhibit different loss-tolerance properties [88], routing metrics must account for resource state type in addition to link fidelity and hop count. Routes optimal for Bell-pair distribution may be suboptimal for W-state distribution across the same topology.
- **Layer 8 (Cognitive Intent Plane):** Orchestration agents must dynamically select between GHZ and W state resources based on application requirements, network topology, and  real-time fidelity telemetry, representing a new class of intent-driven multipartite resource allocation.

#### 5.1.2. Dicke States: Definition, Properties, and Networking Relevance

Dicke states $|D_{k}^{n}\rangle$ generalize the W state to an arbitrary excitation number *k* among *n* qubits, defined as the equal-weight superposition of all *n*-qubit computational basis states with Hamming weight *k* [91]:

(7) |Dkn〉=nk−1/2∑x∈{0,1}n|x|=k|x〉.

The W state is the special case $|D_{1}^{n}\rangle$. Dicke states are symmetric under permutation of qubits, making them natural resource states for protocols involving symmetric or anonymous communication among *n* parties. The entanglement properties of symmetric Dicke states and efficient entanglement witnesses for detecting multipartite entanglement in their vicinity were studied in [92]. Multiparty entanglement in graph states, identifying the utility of symmetric states for quantum secret sharing, was analyzed in [93].

*Experimental Realizations and Deterministic Preparation.*

The first experimental observation of a four-photon Dicke state with two excitations, $|D_{2}^{4}\rangle$, using linear optics and post-selection on SPDC photon pairs was reported in [94], verifying the symmetric entanglement structure of Dicke states. The first deterministic quantum circuits for preparing $|D_{k}^{n}\rangle$ for arbitrary *n* and *k* were proposed in [95], achieving circuit depth O(logn) and gate complexity O(nlogn). Short-depth circuits with linear gate count O(n) suitable for near-term NISQ hardware were subsequently developed in [96].

*Implications for the Quantum-Converged OSI Stack.*

- **Layer 0 (Quantum Substrate):** Dicke state generation requires either deterministic circuit-based preparation on a quantum processor [95,96] or probabilistic photonic preparation via post-selection [94], with  generation fidelity and success probability exposed to higher layers via telemetry APIs.
- **Layer 3 (Network):** Protocols exploiting Dicke states for anonymous transmission or secret sharing require simultaneous distribution to all *n* participating nodes, imposing multicast routing requirements not addressed by standard bipartite entanglement routing protocols [93].
- **Layer 8 (Cognitive Intent Plane):** Orchestration agents must select between Bell pair, W state, and Dicke state resources based on the symmetric or asymmetric topology of the requesting application and real-time fidelity telemetry [92].

Table 10 summarizes the key properties of the multipartite entangled states discussed in this section and their relevance to the layers of the Quantum-Converged OSI stack.

## 6. Layer 1: Quantum Physical Layer

**Figure 7:**
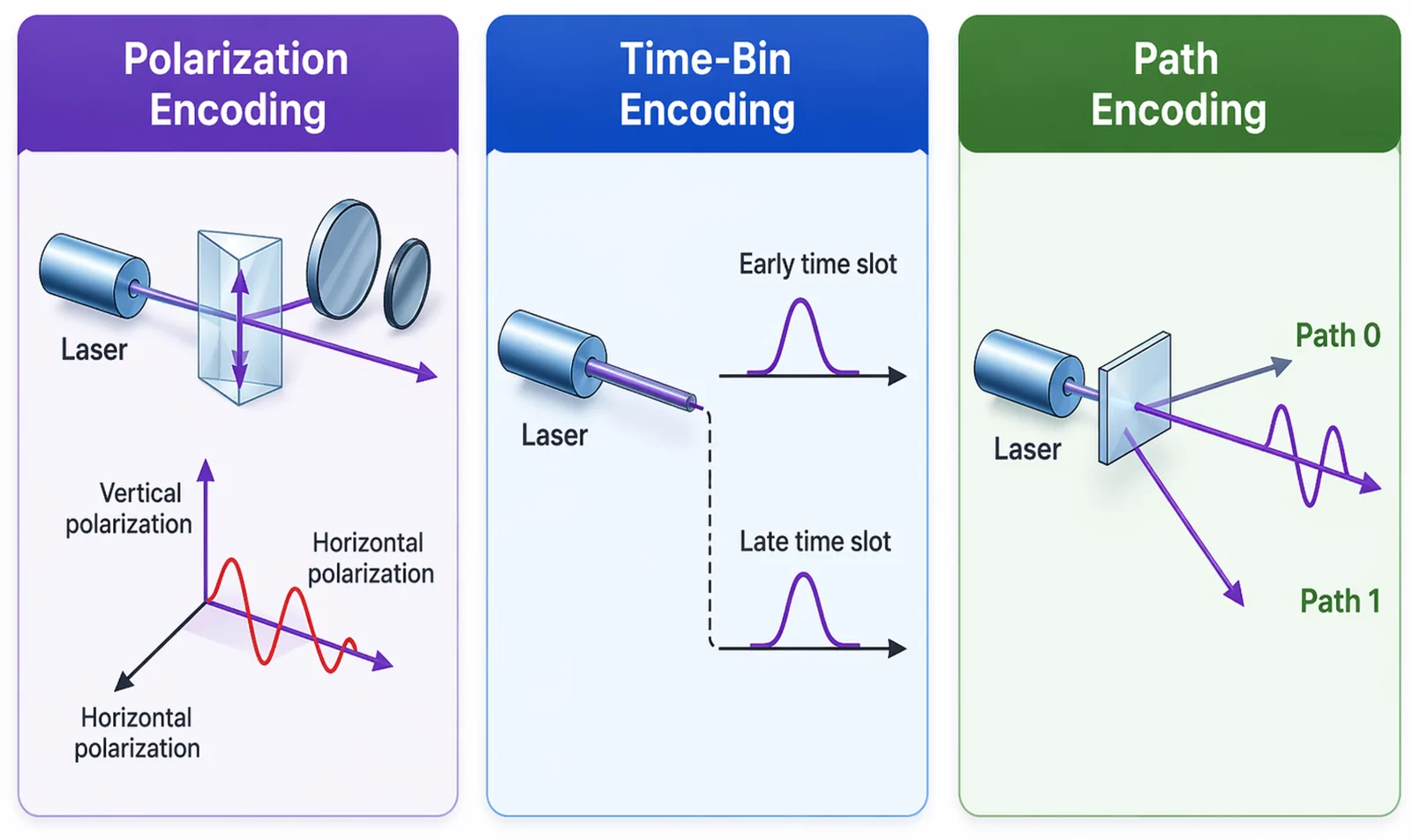
Encoding modalities used in physical-layer quantum transmission, including polarization, time-bin, and path encoding. Polarization encoding is simple but susceptible to drift; time-bin and path encoding offer greater stability over long fiber spans and underpin MDI-QKD implementations. Each modality is contrasted against qubit medium, operating band, and error-control method in Table 11. Source: summarized from [101,102].

Layer 1 in the Quantum-Converged OSI architecture governs quantum signal transmission across diverse media—fiber, free-space, chip-scale, and satellite links—interfacing directly with the entanglement substrate (Layer 0). While Layer 0 generates and stabilizes entangled states, Layer 1 manages their propagation via encoding, modulation, and hardware transduction across optical and matter qubit systems. Quantum-specific challenges such as state fragility, probabilistic propagation, and measurement-induced collapse differentiate this layer from classical physical layers. Transmission technologies include optical fiber, satellite–ground free-space optics, photonic integrated circuits (PICs), chip buses, and waveguides [80,85,86]. Key components—quantum repeaters, frequency converters, and electro-optical modulators—support multiplexing, fidelity maintenance, and routing. Reconfigurable Intelligent Surfaces (RIS) are emerging as programmable metasurfaces that enhance entanglement routing and suppress decoherence under dynamic conditions. While extensively used in classical wireless systems [97,98], their adaptation for quantum contexts shows promise in steering photonic wavefronts and maintaining polarization [99]. This section outlines encoding strategies, quantum sources, waveguide substrates, and RIS-enhanced transmission. Table 11 summarizes key physical-layer protocols such as BB84, E91, MDI-QKD, and satellite-based systems like *Micius* [46,65,86,100]. Physical-layer quantum transmission supports several encoding modalities with distinct trade-offs. Polarization encoding, used in BB84 over fiber and free-space links, is simple to prepare but susceptible to polarization drift and requires active compensation. Phase and time-bin encoding offer greater stability over long fiber spans and underpin continuous-variable QKD and Measurement-Device-Independent (MDI) QKD. MDI-QKD, in particular, relocates the Bell-state measurement to an untrusted intermediate node and thereby removes all detector-side-channel vulnerabilities from the security proof, at the cost of requiring high two-photon interference visibility. These modalities are summarized in Table 11 and Figure 7, which contrast polarization, time-bin, and path encoding against qubit medium, operating band, and error-control method. When quantum distribution is implemented over an open free-space channel (e.g., satellite–ground QKD as in Micius), the propagation and encoding of the quantum signal are governed by Layer 1 (Quantum Physical Layer), while the entangled-state generation and teleportation functions reside at Layer 0 (Quantum Substrate). Open-channel impairments such as atmospheric turbulence and photon loss raise the QBER at these layers and are exposed as fidelity and QBER telemetry to higher layers for adaptive routing and session control.

*A. Requirements:* The physical layer of the Quantum-Converged OSI model ensures the coherent transmission of qubits across diverse quantum media—optical fibers, free-space links, photonic chips, and satellite channels—while contending with state fragility, decoherence, and measurement-induced collapse [11,13]. It must mitigate polarization drift, dispersion, and mode mismatch using tailored encoding schemes such as polarization, time-bin, and path encoding, optimized for compatibility with QKD (e.g., BB84), entanglement protocols (e.g., E91), and quantum teleportation [8,9,101,102]. These encoding choices are organized in Figure 7, which links each modality to the physical-layer transmission behavior described here. Environmental resilience is enhanced via dynamic control interfaces and programmable components like Reconfigurable Intelligent Surfaces (RIS), which modulate quantum wavefronts for improved fidelity in turbulent or lossy settings [99]. Layer 1 must also support wavelength multiplexing and Quantum Frequency Converters (QFCs) to align incompatible memory-transmission interfaces [103,104], while exposing fidelity, QBER, and loss metrics to higher layers for routing, session management, and intent-driven optimization [28,80,85,86].

*B. Existing Literature:* The physical transmission of quantum states across a network has been a central focus of quantum communication research since the inception of QKD. Early experimental demonstrations, such as BB84 [8] and E91 [9], laid the groundwork for secure quantum information transfer using polarization-, phase-, and time-bin-encoded photons. These modalities correspond to the encoding families illustrated in Figure 7. These protocols inherently rely on the robustness of physical channels and their ability to maintain quantum coherence over distance. A key milestone in practical quantum communication was the demonstration of entanglement-based transmission over 144 km of free space between La Palma and Tenerife [102]. This experiment validated the feasibility of long-range free-space quantum communication and provided early empirical insight into atmospheric decoherence and photon loss. Similar challenges have been explored in optical fiber-based implementations, where losses become significant beyond 100–200 km due to absorption and scattering, even with ultra-low-loss fibers [101].

Satellite-based quantum communication represents a significant leap in the physical-layer capabilities of quantum networks. The Chinese Micius satellite has successfully distributed entangled photon pairs over 1200 km and achieved intercontinental QKD between ground stations in China and Austria [80,86]. These demonstrations underscore the importance of free-space optics and orbital geometries as part of the physical-layer transmission infrastructure.

A particularly significant advance in physical-layer quantum transmission security is the Measurement-Device-Independent QKD (MDI-QKD) architecture, which relocates the Bell-state measurement to an untrusted intermediate relay, eliminating all detector-side-channel vulnerabilities from the security proof while requiring only conventional laser sources at the two legitimate parties. MDI-QKD is now one of the standard production-grade Layer 1 modalities documented in the QKD network engineering literature [100]. Beyond single-protocol characterization, the field’s understanding of physical-layer security has matured: the survey in [12] provides a comprehensive taxonomy of Layer 1 encoding modalities across fiber, free-space, and satellite–ground media, mapping each to its dominant decoherence mechanism, characteristic error rate, and compatibility with the quantum repeater architectures in Table 12. The quantum internet protocol stack survey [17] further clarifies how physical-layer modality choice propagates upward through the protocol stack, constraining the QEC strategy at Layer 2 and the routing metric at Layer 3.

Moreover, photon generation and encoding technologies have also advanced significantly. Systems based on SPDC remain a primary source for entangled photon pairs [79]. In contrast, quantum dot and NV center systems offer deterministic single-photon emission under cryogenic conditions [78].

PICs and chip-to-chip quantum buses have emerged as scalable alternatives to bulk optics, enabling low-loss, polarization-maintaining, and temperature-stable transmission on planar substrates [105]. Integrated devices allow quantum gates, beam splitters, and phase shifters to coexist on a single chip, facilitating miniaturized quantum networks and localized Layer 1 operations. In addition, QFC has become increasingly critical for enabling inter-device compatibility across disparate operating wavelengths. Techniques have been demonstrated for converting visible photon emissions (e.g., 637 nm from NV centers) into telecom-band wavelengths (e.g., 1550 nm) suitable for long-distance fiber transmission [103,104].

In parallel, the rise of RIS introduces new possibilities for physical-layer optimization in quantum systems. These metasurfaces, typically composed of programmable sub-wavelength elements, can steer and shape quantum wavefronts to suppress multi-path effects, atmospheric scattering, and photon loss [106]. Recent studies have proposed metasurfaces that operate in low-photon and entangled-state regimes, adapting their reflectivity based on photon detection feedback [99]. Simulation environments such as NetSquid and QuNetSim have modeled Layer 1 constraints like link loss, detector efficiency, and timing jitter, thereby providing insight into physical-layer throughput under varying quantum-device conditions [14]. Timing jitter—the temporal uncertainty in single-photon emission and detection—is a key physical-layer impairment that propagates upward through the stack. Excessive jitter misaligns the temporal windows required for Bell-state measurement and entanglement swapping, effectively shortening the usable coherence window and increasing the QBER. Simulation environments such as NetSquid and QuNetSim model Layer 1 constraints including link loss, detector efficiency, and timing jitter, providing insight into physical-layer throughput under varying quantum-device conditions. At the protocol level, jitter tightens the timing tolerances enforced by Layer 2 memory scheduling and Layer 3 multi-hop synchronization.

The authors in [107] provide a concrete experimental basis for these timing constraints, analyzing synchronization in a two-line fiber-optic QKD system with phase-coded photon states and quantifying how single-photon avalanche diode parameters affect time-frame detection probability; their synchronization algorithm, which accounts for photodetector recovery time after photon registration, directly informs the Layer 1 timing tolerances that propagate upward to Layer 2 MAC scheduling.

*C. Technologies and Challenges:* The quantum physical layer is advancing rapidly toward scalable, high-fidelity, and adaptive qubit transmission. Integrated photonic circuits (PICs) based on silicon photonics and lithium niobate enable on-chip quantum state generation, routing, and manipulation with phase stability and polarization preservation, consolidating Layer 1 components like beam splitters and detectors [105]. For long-distance transmission, fiber optics and free-space optics (FSO) are common, though fibers suffer from attenuation and decoherence beyond 100 km, while FSO faces turbulence and weather-induced losses [108]. Quantum Frequency Conversion (QFC) bridges incompatible photon sources and low-loss telecom bands, preserving entanglement across heterogeneous systems [109]. Reconfigurable intelligent surfaces (RISs) offer dynamic control of polarization and phase for quantum signals but must be tailored to single-photon regimes and orchestration integration [99,110]. Superconducting nanowire single-photon detectors (SNSPDs) now achieve high efficiency and low jitter, but require cryogenic environments [111]. Hardware heterogeneity—across wavelengths, synchronization, and gate fidelities—necessitates adaptive protocols and compensation mechanisms [112]. The contrast among polarization, time-bin, and path encodings in Figure 7 also shows why a single physical-layer format cannot serve all quantum media. Finally, exposing Layer 1 telemetry (e.g., QBER, coherence decay, link loss) to upper-layer orchestration requires standardized APIs and middleware for real-time feedback [113].

**Figure 8:**
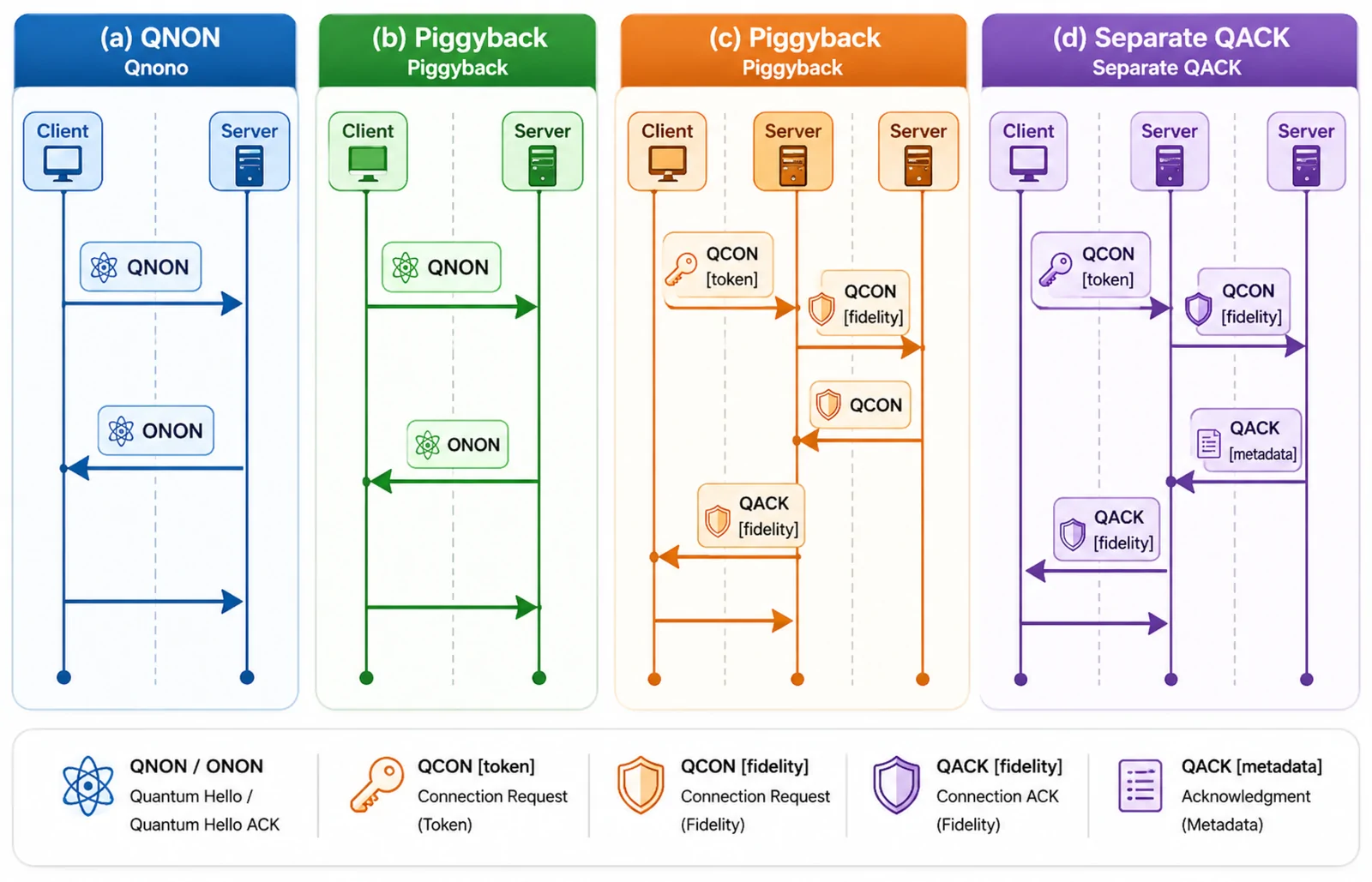
Quantum link-layer operations including MAC arbitration, entanglement feedback, and classical coordination. Entanglement success is probabilistic and governed by decoherence and QBER constraints. Layer 1 QBER and timing-jitter telemetry serve as direct inputs to the MAC arbitration logic, which schedules subsequent entanglement attempts based on coherence-window availability [114,115].

These exported metrics become the direct inputs to the quantum MAC arbitration and feedback flow shown in Figure 8.

## 7. Layer 2: Data Link

**Figure 9:**
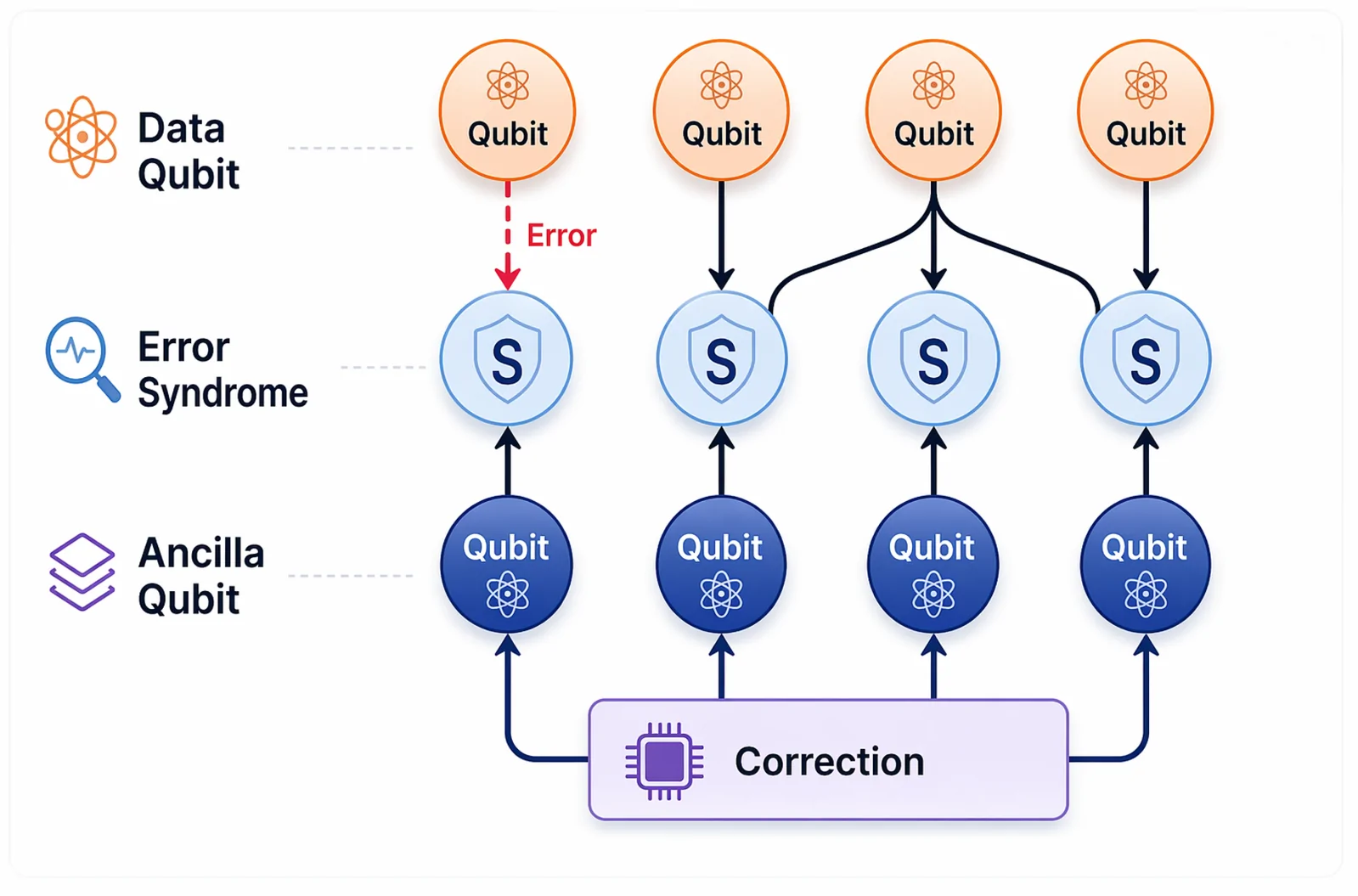
Illustration of link-layer QEC using syndrome-based detection and ancilla qubits for non-invasive error recovery. The syndrome path detects and corrects phase-flip and bit-flip errors while preserving the encoded quantum state, with QEC status exported upward via the Layer 2 telemetry API as input to Layer 3 routing decisions [39,40]. Arrows indicate the direction of information flow: the horizontal path carries the encoded logical qubit, while the branch to the ancilla block carries the extracted syndrome and the resulting correction back to the data path.

**Figure 10:**
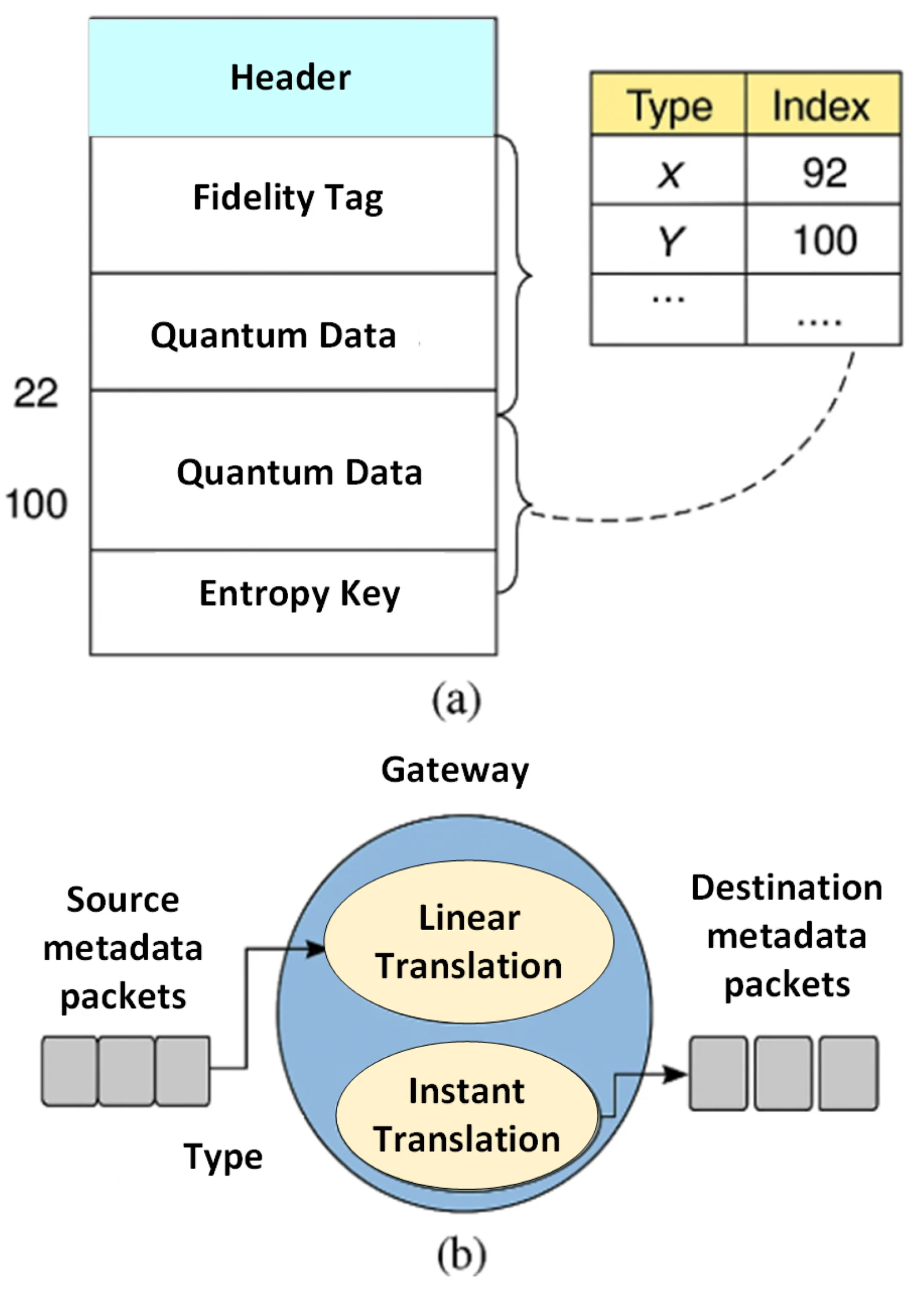
Quantum metadata encapsulation and gateway translation supporting cross-layer telemetry exchange. (**a**) Structure of a quantum metadata frame, comprising a header, a fidelity tag, quantum data payload segments, and an entropy key; the accompanying type–index table maps each payload segment to its type identifier, and the ellipsis denotes further type–index pairs of the same form, which carry no additional information. (**b**) Metadata gateway, in which source metadata packets are mapped onto destination metadata packets either by linear translation, applied in order across a batch of packets, or by instant translation, applied per packet to minimise latency. Layer 2 QBER, memory-age, and entanglement-state telemetry are carried in these metadata fields to Layer 8, which issues reconfiguration commands downward to adjust MAC scheduling, encoding profiles, and entanglement path selection in real time [113,119].

Layer 2 in the Quantum-Converged OSI model governs quantum link access, error resilience, and coherence-aware coordination between directly connected nodes. Unlike classical MAC layers, it must manage quantum-specific constraints such as decoherence, probabilistic entanglement, and non-deterministic transmission success [114,116]. Quantum MAC protocols at this layer dynamically arbitrate entanglement link usage based on real-time fidelity telemetry and coherence thresholds [113,117,118]. As shown in Figure 8, this arbitration depends on both quantum-link outcomes and classical coordination messages. Hybrid quantum–classical signaling is essential, enabling entanglement confirmation, memory coordination, and MAC arbitration via classical acknowledgments [114,115]. A core function of Layer 2 is quantum error correction (QEC), which employs ancilla qubits and syndrome decoding to preserve qubit integrity without violating the no-measurement constraint. The syndrome-based link-layer recovery path in Figure 9 reflects this non-invasive correction model. Modern surface codes and low-overhead QEC schemes are redefining fault tolerance in noisy memory environments [39,40]. Additionally, Layer 2 manages quantum memory scheduling, entanglement buffering, and link-layer handshakes—coordinating qubit release and memory aging policies with higher layers such as routing (Layer 3) and sessions (Layer 5) [116]. Emerging approaches integrate AI-driven MAC control and fidelity forecasting, leveraging entropy metrics, QBER trends, and coherence decay to optimize transmission strategies [113,119]. These predictive feedback mechanisms increasingly tie into orchestration logic at Layer 8 through the telemetry loop illustrated in Figure 10, supporting dynamic, intent-aware control of quantum communication links.

*A. Requirements:* Layer 2 of the Quantum-Converged OSI model functions as the coordination interface between raw quantum transmission (Layer 1) and entanglement-based routing (Layer 3), overseeing channel access, fidelity preservation, and entanglement scheduling. Unlike classical MAC protocols, the quantum data link layer must operate under physical constraints, such as decoherence, the no-cloning principle, and measurement collapse. Fidelity-aware MAC schemes are essential for probabilistically scheduling qubit transmissions based on real-time link quality, qubit readiness, and memory availability [114,117]. Quantum error correction (QEC), using ancilla-based surface or topological codes, must counter gate errors and noise without collapsing the quantum state [39,40]. This ancilla-mediated syndrome flow is the same operational pattern depicted in Figure 9. Quantum memory buffers must be synchronized for state storage, lifetime control, and garbage collection of degraded entanglement [116]. Classical coordination remains vital: heralding, acknowledgments, and feedback loops affect synchronization and channel framing [115]. Figure 8 captures this hybrid exchange by separating probabilistic quantum outcomes from the classical messages used to schedule the next link-layer action. The layer must also expose telemetry—QBER, fidelity scores, entanglement aging—to support adaptive routing, session policies, and orchestration [113]. To ensure scalability and hardware independence, Layer 2 should remain modular across quantum substrates, including photonic, ion-trap, and NV center systems, while supporting hybrid QKD, repeater-based routing, and co-located quantum processors.

*B. Existing Literature:* The recent literature has seen rapid development in Layer 2 components for quantum networks, particularly in quantum MAC protocols and QEC. As quantum networking transitions from experimental platforms to early deployments, the reliability of link-layer operations under probabilistic, noisy, and decoherence-prone conditions is a core concern. A major focus has been on the design of quantum MAC protocols that coordinate entanglement distribution and qubit transmission over shared links. Yu and Devitt proposed Q-MAC, a probabilistic MAC protocol that accounts for channel decoherence, memory availability, and entanglement generation success rate [114]. The authors further extended this model by developing an adaptive MAC protocol that dynamically reconfigures link arbitration based on coherence aging and real-time fidelity estimation [117]. A recent survey by categorizes quantum MAC strategies into contention-based, reservation-based, and hybrid approaches, noting the lack of standardization and the need for machine learning-driven arbitration [118].

Quantum memory scheduling and buffering, critical for Layer 2 resource management, has also received attention. The authors proposed fidelity-aware quantum memory management protocols that prioritize qubit refresh and discard policies based on estimated coherence lifetime and teleportation latency [116]. Their results highlight the need to couple memory operations with QEC and routing strategies to prevent fidelity loss due to aging.

A further line of research addresses classical–quantum coordination, which is essential for synchronizing heralded entanglement, link-layer acknowledgment, and syndrome communication. Bhaskar and Roy showed that efficient classical signaling protocols can significantly reduce frame reordering and latency in distributed QKD and teleportation networks [115]. More recently, telemetry-driven MAC and QEC orchestration has been proposed to unify Layer 2 behaviors with upper-layer decision-making. Figure 10 provides the corresponding control-loop abstraction, linking link-layer measurements to orchestration decisions. The authors introduced a telemetry API framework that exposes quantum link-layer metrics—such as QBER, qubit freshness, and memory state—to Layer 8 orchestration agents [113]. This has enabled early implementations of AI-driven fidelity prediction models that adjust entanglement attempts based on historical link behavior [119]. Table 13 compares representative MAC and QEC strategies proposed for Layer 2 implementation.

*C. Technologies and Challenges:* Implementing a scalable, high-fidelity Layer 2 in quantum networks poses substantial architectural challenges. Central among them is the realization of reliable quantum MAC protocols capable of coordinating access to entanglement links under stochastic generation success, latency, and decoherence. Adaptive MAC approaches using entropy-aware feedback and QBER minimization have been proposed [117,118], yet remain simulation-based and lack standardized APIs. *Quantum error correction (QEC)* presents another cornerstone, with current surface and repetition codes requiring minimal overhead but imposing stringent constraints on gate fidelity and ancilla synchronization [39,40]. Figure 9 clarifies why synchronization is central: the syndrome path must detect and correct errors while preserving the encoded state. Memory management is equally critical: physical memories must account for limited coherence times using fidelity tracking, memory aging, and predictive retirement policies [116]. These constraints are compounded by delays in classical signaling, which must synchronize teleportation, syndrome messaging, and link status without inducing memory expiration [115]. Middleware frameworks exposing fidelity telemetry to orchestration agents offer stack-aware MAC control [113], though achieving sub-millisecond responsiveness remains difficult. AI-assisted schedulers that predict decoherence trajectories and reserve proactive transmission windows show promise but remain nascent [119]. Hardware heterogeneity further complicates Layer 2 deployment, necessitating abstraction layers and modular MAC-QEC pipelines to normalize behavior across diverse photonic, trapped-ion, and spin-based substrates. Lastly, security concerns—including rogue entanglement flooding and coherence-based denial-of-service—require the embedding of telemetry-driven scheduling policies and authenticated MAC procedures within federated quantum environments [113,119].

## 8. Layer 3: Network

**Figure 11:**
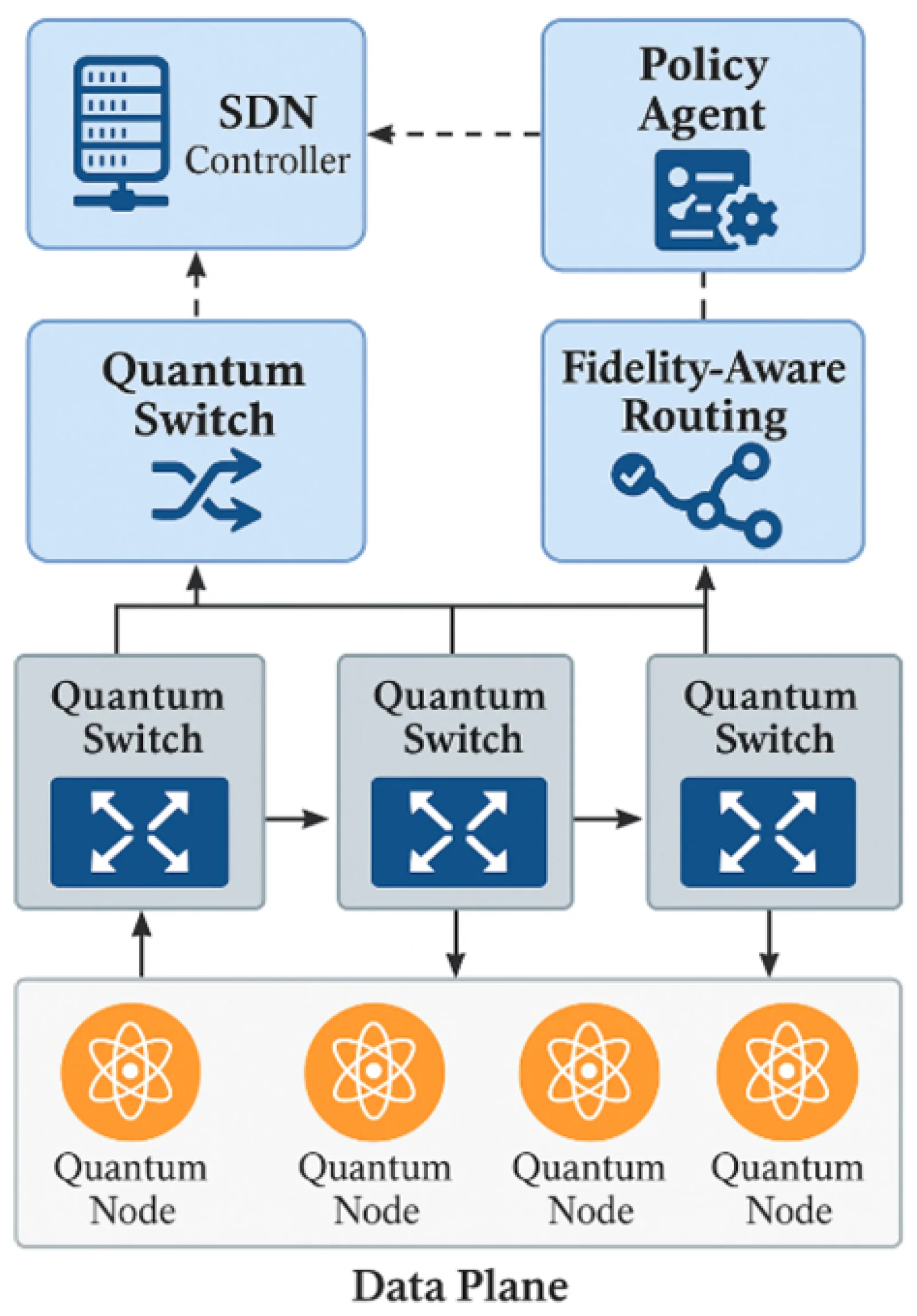
Software-Defined Quantum Network architecture for Layer 3, showing centralized orchestration with fidelity-aware path control and multi-hop entanglement routing. The controller consumes per-link QBER and coherence telemetry from Layer 2 to compute optimal entanglement paths, enforcing routing decisions via programmable forwarding rules at each quantum node. Adapted from [48,113]. Arrows indicate the direction of control and telemetry flow: upward arrows carry per-link QBER and coherence telemetry from the quantum nodes to the controller, and downward arrows carry the resulting forwarding rules back to the nodes.

Layer 3 in the Quantum-Converged OSI model is the network control and entanglement routing layer, responsible for path selection, fidelity monitoring, and qubit forwarding across multi-hop quantum networks. Unlike classical routing, where packets can be duplicated or rerouted with minimal impact, quantum routing must manage fragile, non-clonable qubits whose viability depends on coherence time, memory scheduling, and probabilistic entanglement success [48,120]. The primary function of this layer is to coordinate the establishment and maintenance of entangled links between non-adjacent nodes, enabling quantum teleportation, distributed quantum computation, and end-to-end QKD. A key evolution in this space is the advent of SDQN. Inspired by SDN paradigms, SDQN introduces a logically centralized controller (or cognitive plane at Layer 8) that operates based on real-time fidelity, buffer state, and network entropy conditions [113]. SDQN controllers have been proposed and validated in simulation to dynamically manage entanglement routing [48], but end-to-end deployment across heterogeneous quantum hardware nodes remains a research objective rather than a demonstrated capability. Additionally, Layer 3 must accommodate quantum-aware routing metrics that reflect decoherence rates, link reliability, memory aging, and entanglement fidelity—not just hop count or latency. Multi-path entanglement, probabilistic forwarding, and coherence-time-constrained routing require algorithmic innovations far beyond classical shortest-path protocols [121,122]. Figure 11 illustrates an SDQN architecture where Layer 3 controllers adaptively route entangled links under fidelity and coherence constraints [48,113]. In upcoming quantum Internet deployments, Layer 3 will interface directly with physical-layer telemetry (Layer 1), memory coordination (Layer 2), and orchestration agents (Layer 8), forming the backbone of scalable, programmable, and survivable quantum infrastructures. The following subsections provide a structured analysis of Layer 3’s requirements, supporting literature, enabling technologies, and forward-looking challenges.

*A. Requirements:* Layer 3 in a quantum network stack diverges sharply from classical routing paradigms by managing fragile, probabilistic, entangled links rather than deterministic packet paths. Its core function is fidelity-aware entanglement routing, where routes are selected based on coherence time, QBER, entanglement fidelity, and buffer availability—metrics sensitive to quantum state degradation and non-determinism [120,121]. The layer must also orchestrate entanglement swapping across non-adjacent nodes, synchronizing Bell-state measurements with classical signaling and Layer 2 buffer coordination, while tolerating rerouting due to delays or decoherence [48]. A probabilistic path computation engine is essential, using statistical models or RL rather than traditional shortest-path algorithms to accommodate link instability and coherence-aware constraints [122]. Integration with SDQN (Software-Defined Quantum Networking) controllers allows centralized policy enforcement via telemetry APIs and programmable intent reconfiguration [48,113]. Multi-domain interoperability requires routing support across administrative and technological boundaries (e.g., fiber and satellite), using trust-aware fidelity constraints and entanglement reservations akin to QoS protocols. Finally, Layer 3 ensures session continuity, linking entanglement routing with Layer 5 session establishment and exposing qubit readiness, path scores, and success probabilities to enable predictive binding and QoS-aware applications.

*B. Existing Literature:* Quantum Layer 3 departs from classical routing by managing probabilistic entanglement flows, fidelity decay, and coherence-limited memory. Traditional shortest-path logic fails under these conditions, prompting new paradigms such as Software-Defined Quantum Networking (SDQN) [48], which decouples the control and data planes for fidelity- and intent-aware entanglement routing. The authors in [120] modeled routing as a stochastic BSM-based process. The authors in [121] proposed fidelity-driven path prediction under coherence constraints. Reinforcement learning approaches by the authors in [122,123] enabled adaptive routing via traffic shaping and maximizing teleportation success. The authors in [124] explored hybrid fiber-satellite architectures, highlighting latency–fidelity trade-offs in repeater-assisted routing. The authors in [125] developed a multi-tenant path engine with trust-aware session isolation, while the authors in [113] introduced telemetry-integrated routing APIs supporting real-time reconfiguration. Finally, the authors in [119] advanced the concept of cognitive entanglement routing by combining entropy forecasting with RL-assisted path adaptation.

The entanglement routing literature has been systematized in the comprehensive survey by Abane et al. [126], which classifies protocols across the full spectrum from shortest-path to RL-based and traffic-engineering approaches, and identifies the fundamental tension between coherence-limited memory windows and the computational overhead of optimal path computation. The multi-path entanglement routing work of [120]—which provides the algorithmic foundation for the fidelity-aware multi-path formulation adopted at Layer 3 in the proposed stack—showed that exploiting path diversity across a meshed repeater network yields entanglement-rate gains over a single linear chain, while also allowing multiple user pairs to share repeater resources through multiplexing rather than simple time-sharing. Crucially, the SDQN control framework [48] demonstrates that decoupling the quantum data plane from its control plane enables the kind of real-time fidelity-driven path reconfiguration that Layer 3 of the Quantum-Converged stack requires: the controller observes QBER and coherence telemetry from Layer 2, recomputes optimal entanglement paths using the routing primitives in Table 14, and issues forwarding updates without interrupting active entangled sessions.

*C. Technologies and Challenges:* Deploying Layer 3 in quantum networks introduces architectural challenges distinct from classical routing. Unlike deterministic classical links, quantum paths are time-variant and probabilistic, requiring fidelity-aware routing algorithms that adapt to qubit coherence, memory freshness, and stochastic entanglement success [120,121]. Classical protocols like link-state are unsuitable because they assume static metrics, necessitating real-time route recomputation as fidelity degrades. Another complexity is entanglement swapping orchestration, which demands precise timing, classical feedforward, and synchronization between Layer 2 and Layer 3 to prevent fidelity collapse during swap errors [124]. While SDQN offers a flexible control model, its control planes are limited by the lack of telemetry APIs that expose quantum metrics such as coherence decay and memory utilization [48,113]. Centralized orchestration may exceed coherence windows, necessitating edge-level delegation. Scalability also remains unsolved—entanglement path complexity grows combinatorially, and RL-based routing agents, though promising, suffer from slow convergence and limited real-time usability [122,123]. Hardware heterogeneity further complicates routing: differing qubit types and memory characteristics demand platform-normalized metrics via a hardware abstraction layer [125]. Layer 3 must also address security, including quantum denial-of-service (QDoS) threats that exploit scarce entanglement or memory, a problem currently lacking standard mitigation policies [125]. Finally, hybrid terrestrial-satellite networks introduce cross-domain timing, Doppler correction, and dual-mode routing requirements that challenge unified Layer 3 design [124].

## 9. Layer 4: Transport

Layer 4 of the Quantum-Converged OSI model governs the establishment, maintenance, and termination of quantum sessions across distributed nodes, ensuring entanglement coherence and fidelity without violating the no-cloning theorem or inducing quantum measurement collapse. Unlike classical transport protocols, which rely on retransmission and buffering, the quantum transport layer must manage probabilistic entanglement resources via fidelity-aware session binding, timeout negotiation, and temporal qubit allocation. It handles handshake coordination, entanglement-use negotiation, and secure session termination for services such as QKD and teleportation. Flow control mechanisms must operate without buffering, enforcing dynamic scheduling and qubit reservation through tight integration with Layer 2 and Layer 3 via telemetry and signaling. To abstract hardware volatility, Layer 4 remaps entanglement paths in real time, adapting to route failure, decoherence, or memory expiration using inputs from cognitive agents in Layer 8. Emerging techniques include teleportation protocol wrappers, qubit flushing policies, and entanglement time budgeting to maintain upper-layer service continuity under volatile physical conditions.

**Figure 12:**
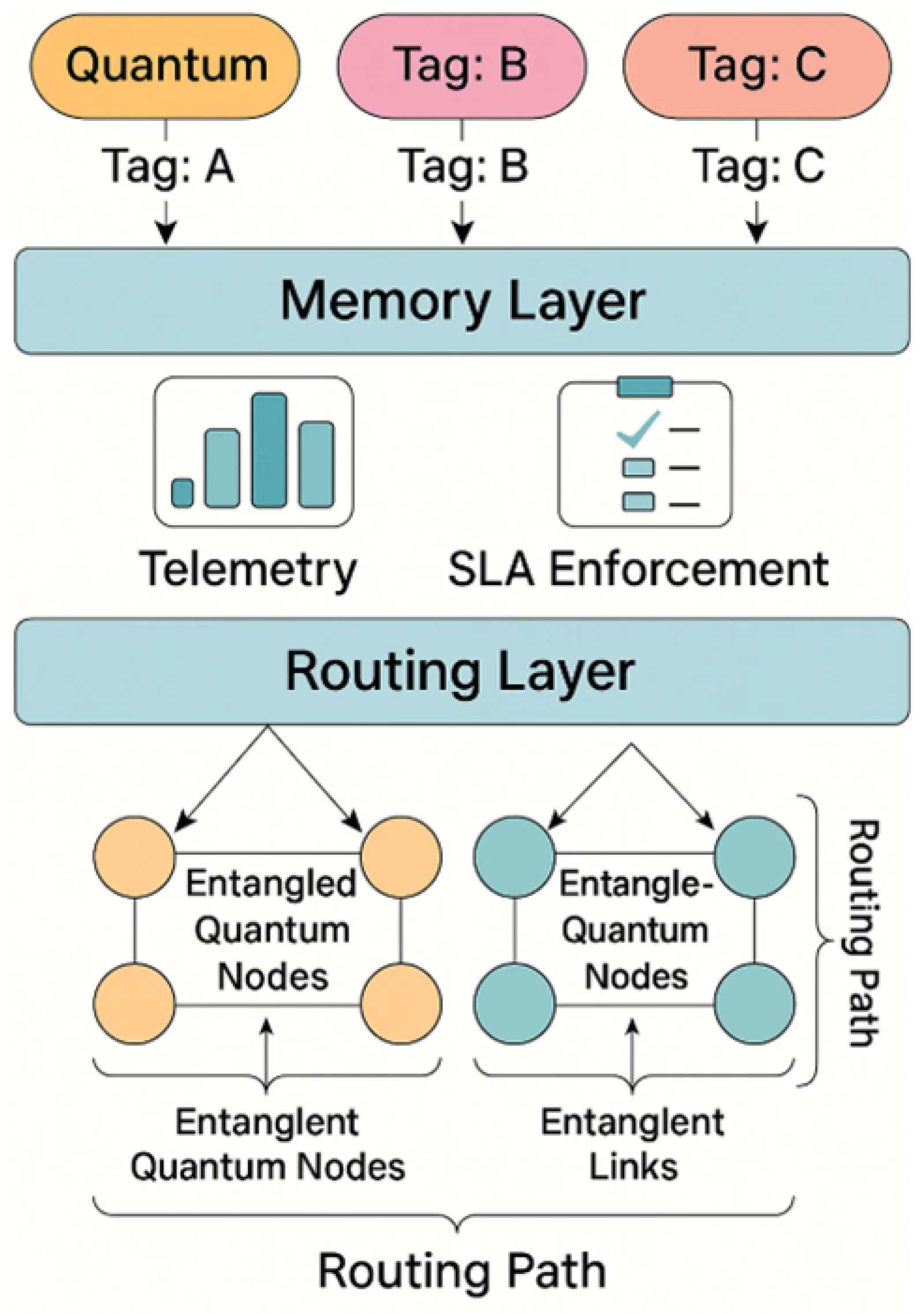
Concurrent quantum sessions multiplexed over shared Layer 2 memory buffers and Layer 3 routing fabric. Each session is tagged with a unique identifier and fidelity scope to prevent entanglement conflict and information leakage across flows. Layer 4 transport coordinates coherence budgets per session, and Layer 8 enforces SLA-aware separation via telemetry-driven admission control [113,130].

*A. Requirements:* Layer 4 of the Quantum-Converged OSI model supports reliable, session-aware quantum communication under fidelity and coherence constraints, replacing classical mechanisms like retransmission and acknowledgments with quantum-native primitives. Core functionality includes quantum session control, enabling fidelity-aware handshakes that negotiate entanglement policies, timeout bounds, and consumption strategies, while coordinating with Layer 3 routing and Layer 2 buffer states [19,127]. The layer also provides flow control mechanisms tailored to lossy, non-clonable quantum links, managing pacing windows and coherence budgets to avoid state collapse during transmission [121]. Fidelity-aware adaptation is vital, requiring the transport layer to respond dynamically to metrics such as QBER, memory expiry, and entanglement degradation by renegotiating session parameters or rerouting paths [113]. To ensure resilience, the layer supports session abstraction and continuity, decoupling logical quantum sessions from underlying entangled paths and reassigning them as link quality fluctuates. Additionally, it enforces application-level QoS constraints—such as minimum fidelity thresholds and teleportation latency—which trigger orchestration feedback when violated [19]. Finally, Layer 4 must implement multiplexed session coordination, allowing multiple quantum applications to share entanglement pools while preserving task isolation and coherence integrity, as later summarized in Figure 12.

*B. Existing Literature:* The development of transport-layer protocols for quantum networks is a recent but critical area of research. Traditional TCP/IP-based models are inadequate in quantum contexts, where retransmission, buffering, and packet duplication are physically impossible due to the no-cloning theorem and coherence sensitivity. As a result, researchers have proposed transport-layer architectures centered on quantum session control, entanglement scheduling, and fidelity-aware flow policies.

One of the first formal models of quantum session abstraction proposes a session-aware protocol that coordinates entanglement acquisition and maintenance under fidelity and coherence constraints [127]. Their work emphasizes decoupling logical session states from physically entangled links, enabling applications to operate transparently even as fidelity degrades or paths are re-allocated. Schoute and Wehner proposed a layered framework for quantum transport services, including teleportation session setup, memory-aware qubit flushing, and fidelity-constrained delivery guarantees [19]. They define transport-layer contracts that allow applications to request specific coherence durations, minimum entanglement fidelity, and path continuity, all while leveraging telemetry feedback from lower layers.

The authors in [121] contributed to the conversation from a routing-adjacent angle, introducing fidelity-aware timing and pacing constraints for teleportation flows over unreliable links. Their results showed that link-layer metrics—such as entanglement age and qubit freshness—must be explicitly considered at the transport level to prevent session violations or fidelity loss.

Another line of research has addressed teleportation-lifecycle-aware transport design. The authors modeled quantum transport as a lifecycle system, where each qubit pair undergoes entanglement generation, fidelity verification, teleportation usage, and measurement collapse [128]. Their framework suggests buffering pre-verified Bell pairs and integrating application-level fidelity policies directly into session controllers.

The authors extended these ideas by simulating transport-layer error flushing policies, where qubits that fall below fidelity thresholds are automatically released or redirected to lower-priority tasks [129]. Their simulation study highlights the risk of fidelity “deadlocks,” in which low-quality qubits block session progress unless actively flushed or replaced. Emerging work by the authors explores multiplexed quantum session scheduling across shared memory pools and routing interfaces, proposing tagging-based qubit isolation for concurrent transport flows [130]. This design aligns with Figure 12, where tagged sessions share quantum resources while remaining logically isolated. Their scheme supports prioritization, inter-session handoffs, and coordination of teleportation results.

**Figure 13:**
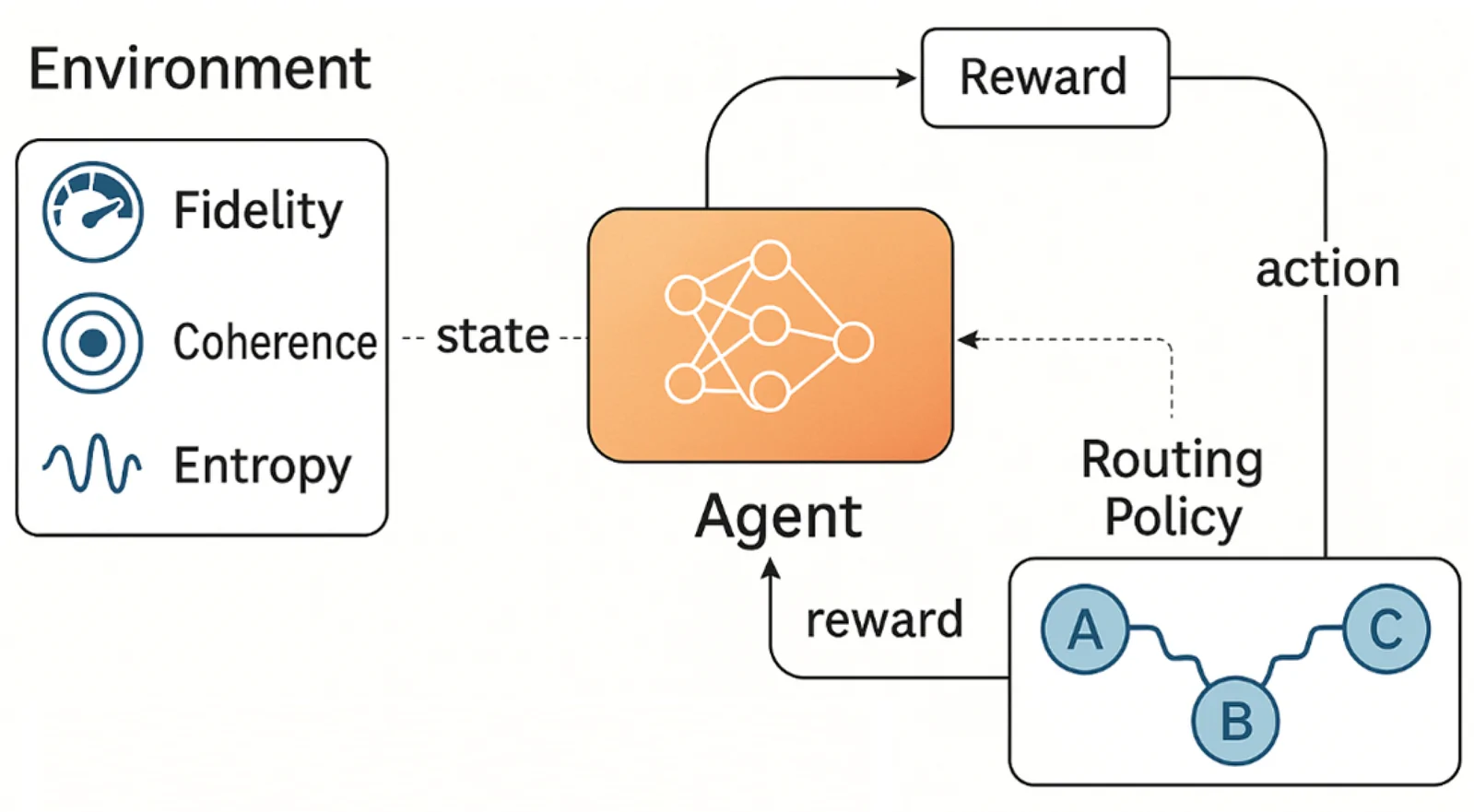
RL-assisted quantum routing model. The agent observes fidelity, coherence, and entropy telemetry from Layers 1–2 and learns optimal entanglement path selections via reward signals tied to teleportation success probability and session continuity. The routing entropy metric $S_{r}=-\sum_{i}p_{i}\log p_{i}$ quantifies path-selection balance and is consumed by Layer 8 for load-balancing decisions. Based on [119,122].

*C. Technologies and Challenges:* Designing a scalable quantum transport layer requires addressing the intrinsic fragility of entanglement, the irreversibility of qubits, and the non-deterministic behavior of links. A core challenge is providing session-level abstraction over volatile entanglement paths, requiring dynamic rerouting and resource renegotiation without interrupting logical flows [19,127]. Unlike classical buffering, quantum flow control must operate under no-cloning constraints, with delivery governed by timing windows and coherence budgets [113,129]. Transport protocols must tightly coordinate with Layer 2 memory aging and Layer 3 fidelity forecasting to manage teleportation timing and session lifetimes [121,128]. Real-time telemetry integration remains a hurdle, as metrics like QBER, qubit freshness, and path uptime must drive sub-millisecond transport decisions [113]. Multiplexed session scheduling further complicates flow isolation and fairness across shared quantum resources [130]. Figure 12 emphasizes this challenge by placing concurrent sessions over the same memory and routing fabric, requiring explicit tagging and SLA-aware separation. Finally, heterogeneous hardware support for transport primitives such as fidelity-aware reservation, teleportation callbacks, and memory signaling demands standardized abstraction layers for interoperability. Figure 13 visualizes dynamic control under link volatility to preserve teleportation viability [113,129]. Table 15 summarises the quantum transport-layer strategies discussed above together with their principal limitations.

## 10. Layer 5: Session

**Figure 14:**
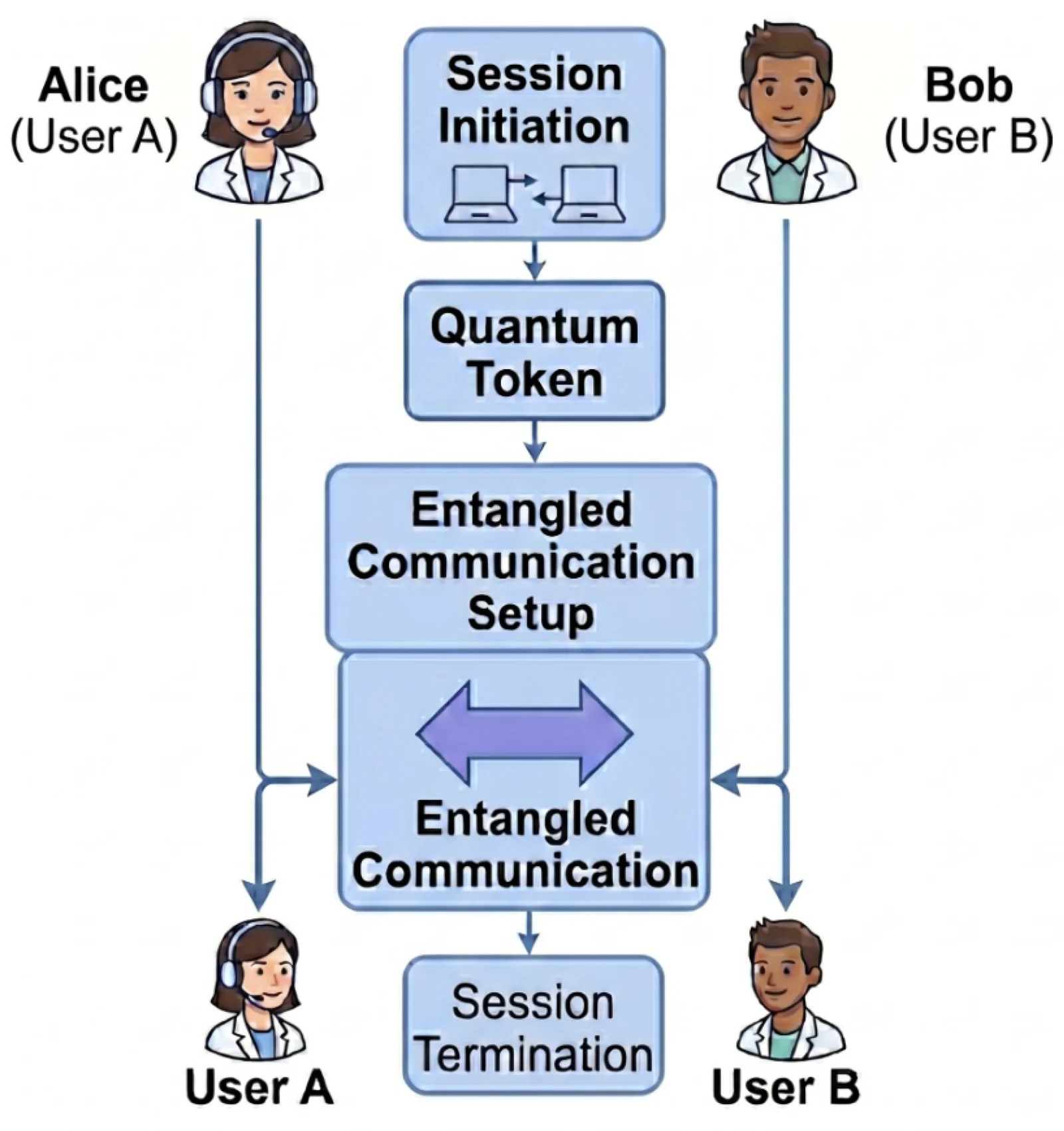
Session-layer role negotiation across trust domains. The sender, verifier, and receiver exchange authenticated metadata to bind entangled states with session logic, enforcing fidelity-scoped contracts and identity–role consistency across Layer 5 session boundaries.

Layer 5 in the Quantum-Converged OSI model serves as the orchestration hub for managing logical quantum sessions between distributed applications over physical and virtual quantum links. Unlike the transport layer, it aligns session logic with service roles, identity resolution, and policy agreements. Core responsibilities include quantum service binding—mapping Layer 7 intents (e.g., QKD, quantum sensing) to entanglement resources—and executing handshake protocols that enforce session type, role, and fidelity requirements. The session layer supports cross-domain coordination by resolving identity bindings and enforcing semantic agreements across heterogeneous infrastructures [131,132]. It also ensures fidelity-aware session-state maintenance, responding to decoherence, QBER drift, or routing failures via renegotiation or termination triggers, in coordination with Layer 4 and Layer 8. Recent advances include Quantum Session Managers (QSMs), identity-aware qubit tagging, and cross-platform brokers that facilitate role propagation and trust across federated domains. These capabilities are critical to ensuring resilient and adaptive quantum workflows in increasingly dynamic network environments. These role-binding interactions are illustrated in Figure 14, where authenticated metadata connects sender, verifier, and receiver responsibilities to the underlying entangled session.

*A. Requirements:* The session layer in a quantum-converged stack abstracts low-level entanglement flows into application-contextualized service sessions, managing their initiation, maintenance, and termination while adhering to coherence and fidelity constraints. A primary requirement is quantum service binding, mapping high-level tasks like QKD, QSMC, or delegated computing to entangled sessions with fidelity guarantees and time-bound execution, in alignment with orchestration policies from Layer 8 [127,133]. The layer must also enable identity-aware session coordination, assigning roles (e.g., sender, verifier) based on authenticated identity profiles and maintaining quantum contract integrity throughout the session, as reflected in Figure 14 [131]. Support for cross-domain session control is essential to orchestrate multiparty interactions across heterogeneous networks, enforcing synchronized protocol states and shared trust policies [133,134]. Furthermore, fidelity-scoped session contracts must be enforced using telemetry from lower layers, triggering rebinding or teardown when entanglement quality degrades below SLO thresholds [113]. Layer 5 also exposes session lifecycle APIs—such as create, pause, resume, and teardown—while enabling adaptive transitions, such as migration or role reassignment. Finally, it supports concurrent session isolation and tagging, ensuring that entanglement resources and participant roles are uniquely scoped per session, avoiding interference and leakage in shared quantum infrastructures. Table 16 summarises the session-layer role-binding models and their operational characteristics.

*B. Existing Literature:* Recent advances in quantum networking have catalyzed interest in session-layer architectures that abstract entanglement flows into secure, role-aware, and service-consistent quantum sessions. The session layer has been reconceptualized not simply as a communication convenience, but as a crucial coordination and binding layer for application-level tasks such as QKD, delegated computing, and distributed consensus. Their approach defines sessions as dynamic, role-scoped containers that include fidelity thresholds and validity windows to support advanced applications like teleportation-as-a-service [127]. The authors proposed one of the first cross-domain session orchestration models, highlighting the problem of heterogeneous session binding across administrative boundaries. They introduced Quantum Session Descriptors (QSDs) to capture role assignments, fidelity contracts, and policy constraints, which can be exchanged and verified across domains for secure session instantiation [133].

The authors focused on role-aware coordination, showing that many multiparty quantum protocols—e.g., GHZ-based voting or conference keying—require persistent and verifiable role assignments (e.g., leader, sender, verifier). This role structure matches Figure 14, where the sender, verifier, and receiver are explicitly bound to the session metadata. Their proposed session layer implements a quantum role registry and session authorization logic to ensure identity–role consistency [134]. Rao and Kim addressed the challenge of identity resolution and secure session admission. They proposed an identity-based session negotiation protocol where node identifiers are cryptographically bound to session requests, ensuring that entanglement-based services are accessible only to authenticated entities with pre-established role profiles [131]. The authors extended the SDQN stack with session-aware telemetry interfaces. Their architecture enables Layer 5 to query fidelity scores, memory state, and link usage history to validate session readiness and make live session migration decisions in response to fidelity degradation [113].

*C. Technologies and Challenges:* Implementing a robust quantum session layer introduces significant challenges due to the volatility of quantum states, fluctuating fidelity, and cross-domain role coordination. A primary hurdle is building session orchestration engines that align entanglement paths, enforce fidelity thresholds, and bind verified identities into coherent session containers [127,133]. Ensuring identity and role consistency across session lifecycles remains difficult, especially in federated environments lacking APIs for cryptographic identity–role assertions or revocable claims [131]. Fidelity-scoped contract enforcement demands integration with Layer 2/3 telemetry and dynamic contract adaptation when QBER, freshness, or coherence constraints are violated [113,128]. Session multiplexing across shared buffers and routing layers introduces risks of information leakage and entanglement conflict, necessitating isolation logic under constrained hardware [130]. Maintaining session continuity through decoherence-triggered rerouting or migration remains an open problem that requires interoperability with Layers 4 and 8 [127]. Cross-domain interoperability complicates negotiation due to heterogeneous session descriptors, identity schemas, and policies [133]. Finally, current session logic is hardware-coupled and lacks programmable APIs, hindering scalable, abstracted deployment over diverse quantum platforms.

## 11. Layer 6: Presentation

Layer 6 in the Quantum-Converged OSI model acts as a semantic bridge between quantum sessions and their informational representations. It performs quantum encoding, decoding, fidelity-aware compression, and format translation, ensuring coherence is preserved while adapting to channel noise, task-specific fidelity requirements, and available qubit resources. Encoding schemes—such as basis rotation, hybrid modulation, and error-tolerant formats—are dynamically selected to match application demands (e.g., QKD, quantum fingerprinting, distributed sensing) and hardware capabilities. The layer also interprets measurement outcomes, applies corrections, and reconstructs logical payloads from collapsed states. In heterogeneous environments, Layer 6 enables cross-platform encoding translation across systems like photonic, ion-trap, or NV-center nodes. The subsections that follow detail the layer’s design criteria, literature foundations, and its evolving role in future quantum internet architectures.

*A. Requirements:* Layer 6 in a quantum network architecture must translate quantum state representations between physical encodings and application-level semantics while minimizing entropy growth and coherence loss. As such, it introduces several non-trivial requirements that differ sharply from classical presentation models. First, the layer must support adaptive quantum encoding based on application context, channel noise, and device capabilities. Encoding schemes—such as dual-rail, time-bin, orbital angular momentum, or hybridized encodings—must be selected to preserve fidelity while remaining compatible with the receiver’s decoding protocol [137]. These encodings must be dynamically negotiated between peers or orchestrated through metadata provided by Layer 5. A second requirement is the implementation of quantum data compression and redundancy minimization. Unlike classical compression, quantum compression must operate without measurement, often leveraging reversible transformations or entanglement-assisted protocols. Therefore, presentation-layer compressors must optimize qubit usage without compromising the integrity of superposition or entanglement states [138]. Another key function of this layer is to provide semantic translation across encoding domains. As quantum networks increasingly span heterogeneous devices, Layer 6 must mediate differences in basis, qubit interpretation, and encoding protocols. This includes translation between photonic and matter-based encodings, time-bin vs. polarization, or logical vs. physical qubit representations [24,139].

Table 17 compares the quantum encoding schemes used at the presentation layer across hardware platforms.

*B. Existing Literature:* Research on the presentation layer in quantum networks is still emerging, but recent studies have begun formalizing the translation, encoding, and compression mechanisms required to support semantically consistent quantum communications. These efforts ensure that quantum state representations can be interpreted accurately across heterogeneous devices and use cases while preserving fidelity. The authors introduced an adaptive quantum encoding framework that dynamically selects encoding bases depending on noise levels and channel conditions. The authors further expanded this line of research by proposing hybrid modulation schemes that combine photonic and matter qubit states using device-specific optimization strategies. Their results support the idea of a presentation layer that acts as a codec between different quantum hardware platforms while preserving superposition and coherence integrity [137]. The authors contributed to the theoretical foundations of quantum compression, exploring entanglement-assisted lossless protocols. Their study highlights that quantum compression at the presentation layer must balance circuit depth and logical qubit count while considering entropy bounds imposed by quantum mutual information [138]. The authors explored the concept of semantic-aware data translation between application contexts. Their presentation-layer system classifies quantum flows (e.g., QKD, sensing, computation) and maps them to encoding schemes and fidelity tolerances suited for each task. Their architecture introduces translation descriptors as metadata that enable interoperability across multi-service platforms [139]. The authors in proposed a modular presentation layer model for quantum networks that separates logical representation from physical encoding.Their architecture supports cross-stack interoperability by defining translation APIs that adapt qubit data for different quantum processors and memory models.

*C. Technologies and Challenges:* Designing a quantum presentation layer requires addressing coherence fragility, qubit non-clonability, and hardware diversity. This layer must support quantum state interoperability, semantic abstraction, and adaptive resource optimization—without compromising entanglement integrity or inducing measurement collapse. A key challenge is the diversity of encoding schemes. Photonic qubits use polarization or time-bin encoding, while solid-state systems rely on spin or charge states. Bridging these requires encoding translation modules or quantum codecs; however, standardized interfaces for such translations remain nascent [24,137]. Dynamic encoding adaptation presents another unsolved problem. As noise, gate fidelities, and decoherence rates vary over time, the presentation layer must dynamically adjust the encoding bases and modulation schemes. Existing adaptive encoding controllers are not yet integrated into telemetry-driven or hardware-agnostic frameworks. Quantum data compression is limited by circuit overhead and measurement irreversibility. Compression mechanisms must respect entanglement boundaries, avoid entropy amplification, and remain coherence-safe. Techniques like entanglement-assisted compression are promising, but they demand ancilla qubits and precise timing [138]. Semantic translation between application-layer goals and physical encodings is underdeveloped. Tasks such as distributed quantum sensing and QKD differ in fidelity needs and measurement logic. Presentation-layer logic must classify flows by semantics and apply context-aware transformations—capabilities not supported in current stacks [139]. Format abstraction and interoperability remain difficult across multi-qubit and hybrid architectures. As sessions span heterogeneous qubit types, encoding boundaries risk fidelity loss or protocol failure unless abstract presentation-layer pipelines are enforced [24]. Additionally, the lack of standardized APIs for encoding control, compression management, and metadata exchange for formats restricts automation and vendor-neutral system design.Without programmable interfaces, presentation-layer tasks remain manually tuned or statically configured. Finally, all presentation-layer operations must be telemetry-driven and fidelity-aware. Encoding and transformation logic must continuously receive inputs from Layer 2 (e.g., QBER) and Layer 3 (e.g., route stability) to ensure adaptive behavior that maintains coherence, optimizes resource use, and avoids degradation.

## 12. Layer 7: Application

Layer 7 of the Quantum-Converged OSI model serves as the interface between end-user services and the entanglement-based quantum infrastructure. Unlike classical networks where applications interact with deterministic, error-corrected channels, quantum applications must operate over coherence-limited, non-clonable qubit resources subject to probabilistic transmission. Layer 7 supports diverse domains—QKD, teleportation, Distributed Quantum Computing (DQC), quantum sensing, and Quantum Federated Learning (QFL)—each with specific fidelity, timing, and session requirements. The cross-domain federation pattern in Figure 5 motivates this application-layer need for domain-aware orchestration. For instance, QKD demands entanglement recycling and error reconciliation, while teleportation relies on real-time Bell-pair coordination and classical feedback. This layer provides declarative, intent-based interfaces (e.g., initQKDSession(), submitQuantumJob(), bindRole()) that express fidelity thresholds, routing preferences, and qubit policies, which are interpreted by Layer 8 orchestrators for telemetry-driven control. Acting as a semantic boundary, Layer 7 mediates between quantum state behavior and classical control logic, enforcing trust and policy models across multi-tenant and federated environments. As quantum services grow in complexity, Layer 7 must evolve to support modular, programmable application architectures that adapt to decoherence, manage entanglement roles, and integrate with centralized or edge-based orchestration planes. The following subsections detail design requirements, service frameworks, and emerging technologies enabling scalable, programmable quantum applications.

*A. Requirements:* The quantum application layer must support services built atop fragile, probabilistic entanglement by managing fidelity constraints, coherence timing, and multi-role execution in coordination with lower layers. Unlike its classical counterpart, it cannot abstract away network behavior but must explicitly handle application-level quantum service intents—such as QBER thresholds, coherence windows, key refresh intervals, and Bell-pair synchronization—communicated to Layer 8 for orchestration and enforced stack-wide [132,140]. It must also expose dynamic, service-specific control APIs (e.g., initQKDSession(), submitQuantumCircuit()) that integrate with session, transport, and presentation logic while supporting extensible metadata [135]. Application-role negotiation is critical: Layer 7 must bind authenticated identities to sender/receiver/verifier roles with exclusivity or delegation constraints [131]. Furthermore, multi-tenant execution requires isolation of memory, entanglement buffers, and fidelity scope, relying on coordination with session tagging and transport controls [140]. Fidelity-aware execution logic enables dynamic task adaptation—such as suspensions or rerouting under fidelity collapse—via real-time telemetry from lower layers [113,136]. Finally, interoperability across quantum hardware platforms must be achieved through the virtualization of qubit control, encoding, and protocol abstractions, ensuring portability and vendor-agnostic deployment across healthcare, UAV, grid, and financial workflows with different quantum-service constraints. Table 18 summarises the principal quantum application classes and their operational parameters.

*B. Existing Literature:* The quantum application layer requires tight integration with the full stack to ensure fidelity-bound delivery and probabilistic coherence of quantum states—far beyond the formatting and session logic seen in classical Layer 7. The authors in [13] laid the conceptual foundation for a global quantum internet, emphasizing role-aware, adaptive applications over noisy entanglement links. Building on this, the authors in [140] proposed a service-oriented architecture that decouples application logic from entanglement management to enable modular, scalable service deployment. The authors in [135] formalized QKD-as-a-Service, using Layer 7 service descriptors to express fidelity, throughput, and session demands across multi-tenant environments. The authors in [132] introduced Teleportation-as-a-Service (TaaS), offering reusable application primitives for teleportation control and fidelity management. The authors in [136] demonstrated how distributed quantum machine learning (DQML) applications depend on Layer 7 role mapping, fidelity telemetry, and entanglement synchronization to support decentralized inference. The authors in [113] extended this model by embedding telemetry-driven APIs into application workflows, enabling real-time fidelity-aware behavior, memory state adaptation, and trust policy enforcement.

*C. Technologies and Challenges:* Implementing Layer 7 in a quantum network stack presents unique technological and architectural challenges that differ fundamentally from those in classical distributed systems. Quantum applications must contend with probabilistic delivery, decoherence-prone entanglement resources, and dynamic fidelity constraints while integrating into a coherent stack-wide orchestration model. A core challenge is the lack of standardized quantum application interfaces and programming models. While classical applications benefit from well-defined APIs and protocol stacks, quantum service definitions remain ad hoc and tied to specific hardware platforms or middleware abstractions. Developers currently lack common primitives for initiating, monitoring, and terminating quantum service sessions—such as requestTeleportation(), submitDistributedCircuit(), or setQKDPolicy()—which hinders modular design and portability across quantum infrastructures [135,140]. Another significant barrier lies in fidelity-aware service orchestration. Quantum applications must make real-time decisions based on coherence windows, entanglement age, and QBER—all of which are time-varying and resource-dependent. Application logic must be coupled with telemetry streams and orchestration control loops, yet current systems lack the fine-grained hooks and abstractions necessary for dynamic fidelity adaptation [113].

Multi-role and identity binding in distributed quantum applications is also challenging. Applications involving teleportation, blind computation, or federated learning must coordinate authenticated identities across roles like sender, verifier, and receiver. However, quantum platforms lack identity-binding protocols at the application level, making role negotiation and inter-domain trust enforcement difficult to manage securely [131,132]. Additionally, the layer also lacks robust support for cross-platform service abstraction. As applications increasingly span heterogeneous infrastructures (e.g., photonic circuits, NV centers, superconducting qubits), Layer 7 must operate independently of encoding schemes, session policies, and device-specific constraints. Without universal abstractions for service semantics and control, developers must hardcode infrastructure assumptions, undermining scalability and vendor neutrality [24]. Another hurdle is the absence of service composability and reusability. In classical systems, microservices can be composed using containers and APIs. Quantum applications lack composable primitives for reusing entanglement workflows, layering quantum jobs, or chaining teleportation operations. The application layer must evolve to provide container-like wrappers that allow quantum services to be assembled, monitored, and migrated [140]. Finally, the lack of stack-aware simulators and development environments limits testing and deployment. Application developers have limited access to simulation tools that reflect stack-wide behavior (e.g., how Layer 3 routing failures affect Layer 7 fidelity guarantees). Without such platforms, debugging distributed quantum applications and verifying fidelity resilience remains infeasible [136].

## 13. Layer 8: Orchestration Cognitive Plane

Layer 8 extends the classical OSI model by introducing a cognitive orchestration plane that governs stack-wide coordination, dynamic service adaptation, and policy enforcement in quantum–classical networks. This layer interprets high-level service intents from Layer 7—such as fidelity constraints, latency targets, and trust domains—and translates them into runtime decisions that reconfigure layers below. Unlike conventional OSI layers, Layer 8 integrates telemetry from quantum memories, QBER monitors, routing engines, and session handlers to drive adaptive control. It enables intent-aware orchestration using AI/ML models to align resource allocations with real-time constraints, supporting centralized, distributed, or federated orchestration across domains. This layer enforces quantum trust policies and SLAs, handling identity resolution, entropy budgeting, and isolation rules. It can, for instance, restrict untrusted qubit flows, adjust QKD rates, or prioritize teleportation paths based on service guarantees. A critical function is closed-loop telemetry feedback, enabling the orchestration plane to dynamically respond to fidelity degradation, decoherence onset, and memory expiration through actions such as teleportation retries, encoding downgrades, or entanglement path switching. This response cycle is directly reflected in Figure 10, where measured degradation feeds back into adaptive control. In hybrid infrastructures, Layer 8 interfaces with classical orchestrators (e.g., Kubernetes) to support scalable, programmable management of quantum workloads in edge–cloud deployments. It is essential for realizing resilient, SLA-aware, and policy-compliant quantum services in 7G+ networks.

*A. Requirements:* Layer 8 serves as the orchestrating and cognitive control plane of the quantum stack, aligning service intent, real-time telemetry, and cross-layer behavior into coherent, adaptive decision-making. Unlike lower layers with discrete functional roles, this layer drives global coordination of resources, policies, and responses. A key requirement is translating intent-driven service descriptions from Layer 7—such as latency, fidelity, and identity constraints—into operational configurations across Layers 1–7 [132,141]. Equally essential is a closed-loop telemetry architecture that ingests fidelity, QBER, memory, and routing data to trigger reconfigurations such as teleportation retries or session pausing [113,121]. The orchestration plane must further support fidelity-aware control logic using AI/ML or rules to prioritize qubit handling, buffer use, and routing under entanglement scarcity [136]. It must enforce policies across administrative domains—handling session isolation, entropy tracking, identity validation, and SLA adherence [48]. Declarative orchestration APIs are also required to register applications, define rules, and monitor network state, similar to Kubernetes descriptors [113]. Finally, robust failover and resilience logic must anticipate fidelity collapse or session expiry by preconfiguring alternative paths and fallback behaviors [121,132].

*B. Existing Literature:* The orchestration and cognitive control plane for quantum networks is a relatively nascent yet rapidly developing area of research. With the increasing complexity of quantum services and infrastructure, recent efforts have focused on building programmable, telemetry-aware orchestration frameworks that mirror the flexibility and automation capabilities of classical cloud-native platforms. The authors proposed one of the earliest formalizations of SDQN. Their architecture separates the control plane from the data plane in quantum networks and introduces programmable orchestration interfaces for entanglement path selection, memory provisioning, and fidelity management. SDQN enables controllers to dynamically configure quantum flows based on network policies and telemetry updates, making it one of the foundational approaches to cognitive quantum networking [48].

The authors expanded on this vision by introducing an intent-based orchestration framework tailored to quantum networks. Their work allows applications to express high-level service objectives—such as latency or fidelity goals—compiled into network-level actions via orchestration policies. This model supports programmable service chaining and policy-driven rerouting in response to fidelity collapse or qubit expiration [141]. The authors presented a practical implementation of a real-time telemetry infrastructure for orchestrating quantum services. Their stack provides APIs for accessing link fidelity, qubit memory state, entanglement usage statistics, and routing metrics. These telemetry feeds serve as inputs for orchestration agents at Layer 8, enabling stack-wide reconfiguration in response to qubit quality drift or resource bottlenecks [113]. The authors explored fidelity-aware orchestration logic that adjusts transport-layer behavior and routing decisions based on QBER and link lifetime predictions. Their fidelity estimation model supports proactive entanglement path rerouting and qubit drop policies that maintain application-level service guarantees under dynamic network conditions [121]. The authors introduced a specialized orchestration framework for TaaS, showing how cognitive control loops can govern Bell-pair provisioning, qubit synchronization, and teleportation retries. Their architecture exposes a declarative API for orchestrators to initiate teleportation sessions, negotiate fidelity requirements, and trigger entanglement reconnections during service degradation [132]. In distributed quantum machine learning, the authors demonstrated the necessity of cognition-enabled orchestration to schedule qubit sharing, gradient poses unique challenges due to entanglement volatility, decoherence, qubit non-clonability, and the lack of unified, age fidelity decay, memory expiration, and entanglement contention while maintaining global model convergence [136].

Table 19 summarises the orchestration and cognitive control functions provided at Layer 8.

*C. Technologies and Challenges:* Building a robust orchestration and cognitive control plane for quantum networks presents unique challenges due to entanglement volatility, decoherence, qubit non-clonability, and the absence of unified fidelity-aware abstractions. Unlike classical SDN, quantum orchestration must act under probabilistic constraints, ingest real-time telemetry, and enforce intent-driven behavior across the full stack. A key challenge is the development of programmable orchestration agents that map Layer 7 intents to Layer 1–7 actions, such as adaptive routing and encoding selection [48], yet current systems lack orchestration APIs and programmable telemetry interfaces [113]. Low-latency telemetry pipelines are also difficult to implement due to limited, non-invasive export capabilities in quantum hardware [121]. In cross-domain contexts, orchestrators must resolve trust and policy conflicts without exposing entanglement metadata [141]. The non-persistent nature of qubit states demands coherence-aware lifecycle management, requiring predictive models to initiate teleportation retries or session downgrades before decoherence thresholds are breached [132]. Cognitive control using AI/ML, including RL and Bayesian models, is essential for dynamic entropy budgeting and entanglement path optimization [136]. Lastly, the lack of stack-aware orchestration simulators prevents realistic validation of control strategies under fidelity degradation, memory exhaustion, and session churn.

## 14. Cross-Layer Design Considerations

**Figure 15:**
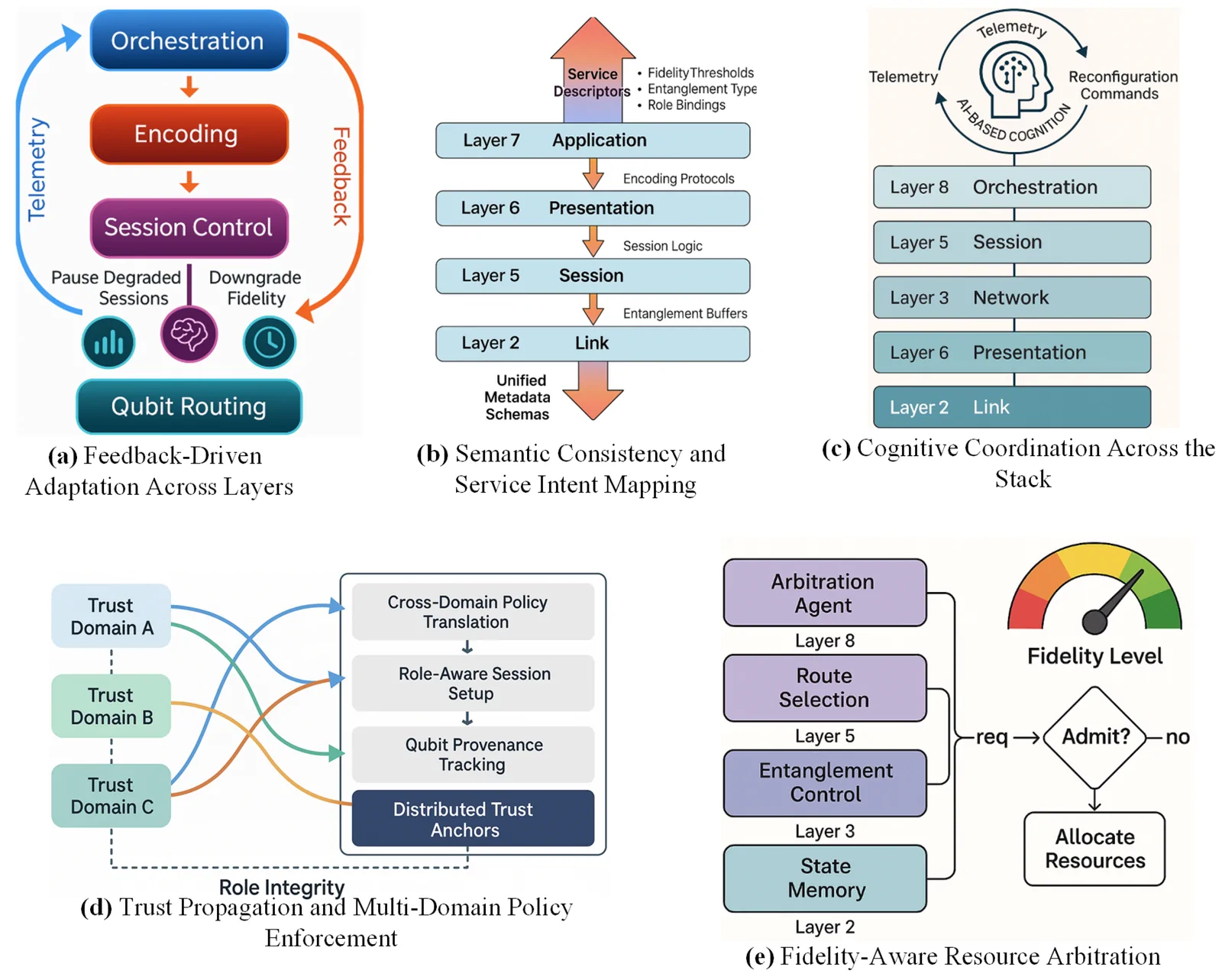
Cross-layer architectural roles in the Quantum-Converged OSI stack. Panel (**a**) shows feedback-driven adaptation where Layer 2 QBER and memory-age telemetry trigger Layer 8 reconfiguration commands. Panel (**b**) shows semantic consistency enforcement, where Layer 7 service descriptors propagate encoding and role constraints down to Layers 2–6. Panel (**c**) shows AI-powered orchestration, where RL and LLM agents at Layer 8 issue path-switching and session-pausing decisions. Panel (**d**) shows trust propagation, where identity tokens flow from Layer 5 down to Layer 2 for qubit provenance tracking. Panel (**e**) shows fidelity-based arbitration, where per-link fidelity scores from Layers 1–3 gate session admission at Layer 5. Within each panel, arrows indicate the direction of information flow between the layers involved, and colours are used only to distinguish layers and functional blocks; they carry no additional quantitative meaning.

While the Quantum-Converged OSI model emphasizes clear modularity across functional layers, real-world implementations demand coordinated interactions to adapt to quantum-specific constraints—especially fidelity degradation, memory expiration, and role-sensitive entanglement policies. Effective quantum networking thus requires intelligent, real-time, and trust-aware cross-layer control. Figure 15 illustrates the key mechanisms of cross-layer coordination in quantum networks. From feedback-driven adaptation (Figure 15a) to semantic consistency enforcement (Figure 15b) and AI-powered orchestration (Figure 15c), the stack enables dynamic and intent-aware quantum communication. Trust propagation (Figure 15d) and fidelity-based arbitration (Figure 15e) further ensure that session integrity and quantum resource viability are preserved across multiple domains and layers.

### 14.1. Feedback-Driven Adaptation Across Layers

Quantum systems exhibit volatile fidelity and coherence lifetimes that fluctuate in response to environmental and system-level factors. To cope, lower-layer telemetry (e.g., QBER, memory age, link uptime) must be continuously fed into higher-layer orchestration logic at Layer 8. These feedback loops allow orchestrators to pause degraded sessions (Layer 5), downgrade encoding fidelity (Layer 6), or initiate qubit rerouting (Layer 3) before services fail [113,121]. This operational view follows the feedback-coupled reference structure in Figure 4, where lower-layer state changes become upper-layer control decisions. This feedback-driven architecture is critical for enabling just-in-time recovery actions—especially in time-sensitive applications like distributed quantum computing and federated sensing, where preemptive adaptation is key to maintaining logical consistency [136].

### 14.2. Semantic Consistency and Service Intent Mapping

Service-layer contracts declared at Layer 7—such as fidelity thresholds, entanglement types (Bell vs. GHZ), and role-to-identity bindings—must propagate consistently across all protocol layers. Misalignments (e.g., mismatched session roles or qubit tagging inconsistencies) can break entangled workflows or introduce silent fidelity collapse [132,141]. To ensure semantic consistency, unified metadata schemas and service descriptors must inform every layer, from Layer 6 encoding protocols to Layer 2 entanglement buffer handling. Future orchestration systems may enforce such mappings via policy validation engines embedded in cognitive control agents [48].

### 14.3. Trust Propagation and Multi-Domain Policy Enforcement

In quantum networks spanning federated or adversarial domains, identity-aware entanglement constraints must be enforced from the application layer down to the physical layers. The multi-domain setting in Figure 5 makes this propagation requirement explicit because each application domain carries different trust, latency, and fidelity expectations. Blind computation, verifiable teleportation, and QKD all require session roles, trust domains, and identity tokens to propagate securely across layers [131,132]. This mandates stack-wide trust enforcement, which includes qubit provenance tracking at Layer 2, role-aware session setup at Layer 5, and cross-domain policy translation at Layer 8. Emerging models propose distributed trust anchors and entanglement ACLs to maintain role integrity in hybrid service scenarios [141].

### 14.4. Cognitive Coordination Across the Stack

Future orchestration layers will incorporate AI-based cognition that integrates telemetry from all layers to forecast entropy depletion, predict session failure, and recommend policy reallocation [136]. These systems will dynamically reconfigure routing paths, encoding strategies, and session priorities based on real-time network conditions and long-term performance learning [121]. Such cognitive loops must operate in stack-aware simulators to safely test adaptation logic under dynamic quantum constraints. This will unlock closed-loop orchestration where decisions propagate downward as reconfiguration commands, and telemetry flows upward for model refinement [113].

Table 20 summarises the cross-layer functional dependencies discussed in this section.

## 15. Enabling Technologies

**Figure 16:**
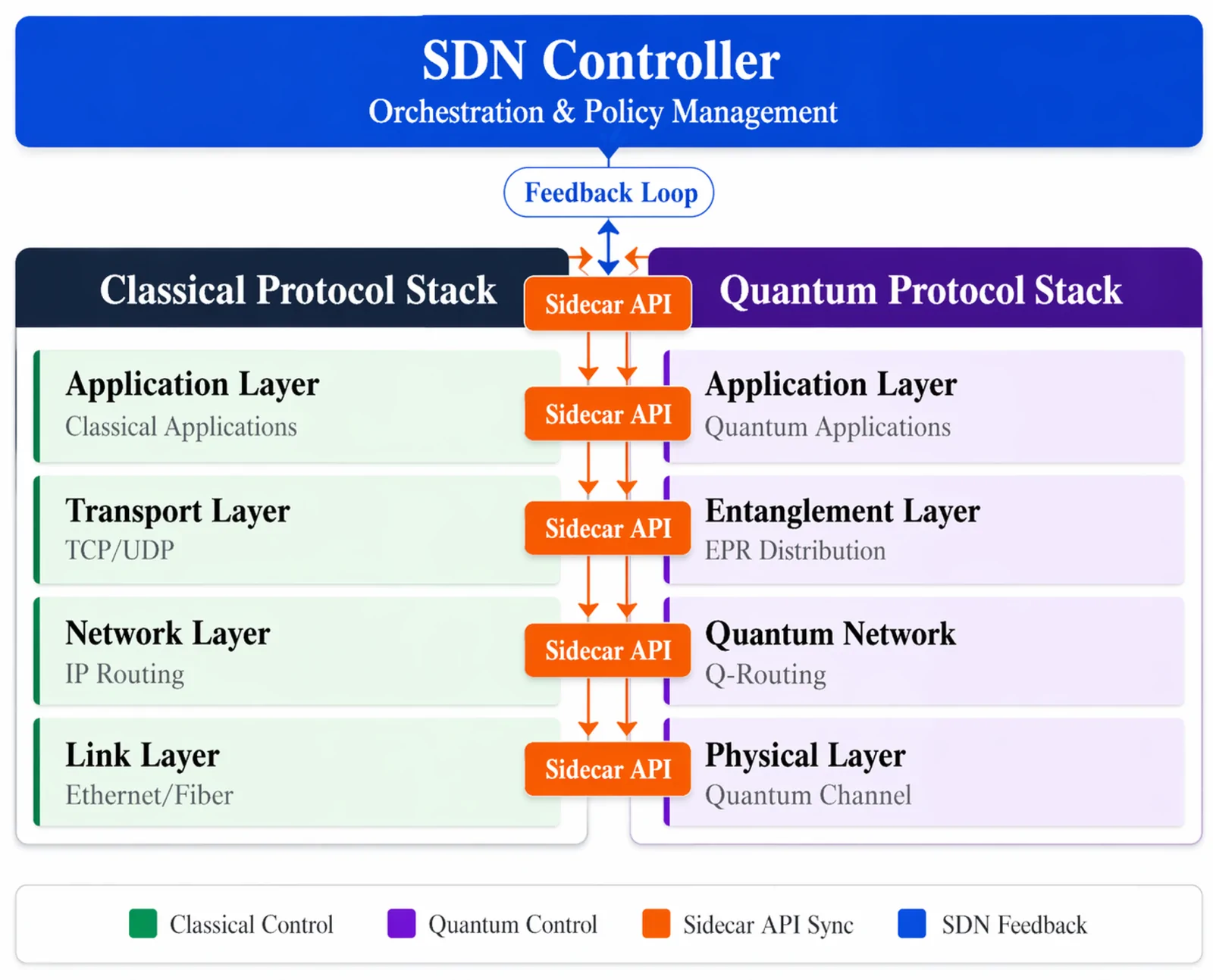
Hybrid quantum–classical architecture showing parallel protocol stacks and sidecar APIs for interoperability. The sidecar APIs synchronize classical SDN control logic with quantum entanglement management, exposing fidelity, QBER, and coherence telemetry across the stack boundary to enable unified orchestration at Layer 8. Arrows indicate the direction of message exchange between the classical and quantum protocol stacks through the sidecar APIs.

The realization of the proposed quantum-converged OSI stack hinges on key enabling technologies that function collectively across layers to support fidelity preservation, coherence control, semantic translation, and intent-based orchestration. QML and LLMs serve as adaptive orchestration agents, translating user intent into real-time stack reconfigurations and predicting qubit behavior based on coherence and fidelity metrics [142]. At the physical layer, RIS dynamically steers the propagation of quantum signals, mitigates polarization drift, and optimizes entanglement channels [143]. For secure communication, QKD is foundational but increasingly integrated with PQC to ensure cryptographic robustness under noise and range constraints [144]. Blockchain infrastructures further enhance trust by enabling decentralized identity, quantum token auditing, and secure metadata coordination, especially within federated QKD deployments [145]. Validation of these technologies is enabled by simulation environments such as NetSquid [14], QuNetSim [15], and QuISP [52], which model decoherence, entanglement routing, and cross-layer protocol behavior. These platforms support testing of entropy propagation and stack efficiency under realistic noise models. Figure 16 illustrates dual-stack coexistence using sidecar APIs that synchronize classical and quantum control logic under SDN-like feedback loops.

### 15.1. Quantum Hardware and Physical Interfaces

The hardware layer forms the physical substrate of quantum communication, underpinning qubit realization, entanglement distribution, and signal fidelity. Photonic qubits dominate long-distance links due to their resilience to decoherence and compatibility with fiber. Quantum dots as single-photon sources [146] and high-efficiency Superconducting Nanowire Single-Photon Detectors (SNSPDs) [68] enable high-precision metropolitan and satellite quantum channels. Quantum repeaters extend communication range via entanglement swapping, with memory-buffered and satellite-assisted designs [58,147]. Hybrid spin-photon interfaces [148] further enhance matter–light integration. Reconfigurable Intelligent Surfaces (RIS) offer dynamic entanglement tuning under non-line-of-sight or turbulence [106,149], enabling coherent urban and satellite QKD links. These hardware innovations are pivotal for the evolution of the OSI stack, guiding protocol-layer decisions through their fidelity, tunability, and preservation of coherence. Table 11 summarizes representative physical-layer protocols such as BB84, E91, MDI-QKD, and satellite-based QKD via Micius [46,65,86,100].

The quantum repeater is the single most critical enabling technology for a global quantum internet, and the Azuma et al. review of quantum repeater architectures [20] provides the field’s authoritative treatment of the hardware trade-offs that govern the Layer 0/Layer 1 boundary in the proposed stack. That review identifies three generations of quantum repeater, differentiated by their error-correction strategy: first-generation repeaters based on entanglement purification and atomic-ensemble quantum memories—the DLCZ protocol [74]—whose practical operational constraints were quantified in the review by Sangouard et al. [75]; second-generation repeaters that combine quantum error correction with efficient photon-matter interfaces; and third-generation all-photonic repeaters. The coherence-time constraints, entanglement generation rates, and error-correction overheads differ significantly across these generations and must be reflected in the telemetry primitives exported by Layer 0 to Layer 1 in Table 8. The stack designer must therefore choose, at the Layer 0/Layer 1 interface, which generation of repeater is assumed, since this choice determines the minimum qubit memory coherence time, the maximum permissible multi-hop latency, and the error-syndrome overhead that the Layer 2 MAC scheduler must absorb.

### 15.2. Quantum–Classical Interface Protocols

The integrity and scalability of quantum networks depend on robust quantum–classical interface protocols that harmonize quantum mechanisms with classical control and transport layers. A key strategy is to integrate PQC with QKD, where lattice- and code-based PQC schemes provide fallback security in the event of QKD disruption [149,150]. Hybrid Software-Defined Networking (SDN/Q-SDN) architectures further enhance integration. In these systems, classical SDN controllers are paired with quantum-aware control planes that dynamically manage entanglement routing and coherence-sensitive orchestration [24]. Blockchain technologies have also emerged as interface enablers, offering decentralized trust and access control across quantum–classical boundaries. Distributed ledgers track QKD session states, verify key lifecycles, and secure API transactions [151,152].

### 15.3. Quantum Software Frameworks

As quantum networking advances toward layered architectures, software frameworks and simulation platforms have become indispensable for protocol development, validation, and stack optimization. Among these, NetSquid, QuNetSim, and QuISP have emerged as leading discrete-event simulation tools. NetSquid supports noise modeling, entanglement queuing, and teleportation delay analysis at various stack layers [14]. Similarly, QuNetSim, written in Python 3 (version 3.7 or later), provides a modular, pedagogically oriented platform for quantum MAC protocols, session initiation models, and routing logic [15]. Meanwhile, QuISP uses a C++-based back-end tailored to full-stack simulation, including physical-layer noise, link-level fidelity tracking, and hybrid classical–quantum channel dependencies [52]. In parallel, development kits like Qiskit, Cirq, and Amazon Braket enable programmers to design and deploy quantum algorithms in hardware-agnostic environments. These SDKs support application-layer integration with OSI layers 6–8, including quantum data compression, API abstraction, and teleportation-based conferencing. The authors in [69] introduced Qiskit as a modular suite with transpiler access and quantum circuit composers.

### 15.4. AI and LLM-Assisted Orchestration

With quantum networks becoming increasingly dynamic and complex, AI-driven orchestration—particularly through LLMs and QML—is emerging as essential for maintaining fidelity, coherence, and adaptive control. LLMs like GPT-4 and domain-specialized variants operate at the Cognitive Plane (Layer 8), translating user intent into protocol configurations and interpreting network telemetry (e.g., fidelity, decoherence) for real-time reconfiguration [142]. At the lower layers, RL and supervised QML models optimize routing, MAC arbitration, and entanglement scheduling under noise and coherence constraints, as shown RL-based gate optimization for superconducting devices [56]. Generative QML models further simulate entanglement dynamics and the impact of collapse [153], enabling predictive orchestration loops in which LLM policy agents are validated through coherence forecasting. This integration marks a paradigm shift toward intent-driven, AI-enhanced stack coordination across hybrid classical–quantum infrastructures. It is important to note that the integration of LLMs into quantum network orchestration remains at an early and largely conceptual stage. Current proposals, outline intent-translation frameworks in which LLM agents parse service-level objectives and map them to protocol configurations. However, formal protocol definitions, latency bounds, and interference analysis for such systems are still outstanding research problems that require experimental validation before deployment in real quantum infrastructure.

### 15.5. AI/ML and Quantum Intelligence Integration

As quantum networks shift toward coherence-sensitive, probabilistic architectures, classical control protocols falter, prompting AI-driven orchestration via Quantum Machine Learning (QML), Large Language Models (LLMs), and Reinforcement Learning (RL). QML enables real-time modeling of decoherence, entanglement optimization, and adaptive routing under dynamic noise conditions [153]. LLMs, trained on quantum protocol grammars, operate in Layer 8 as intent translators and semantic agents—mapping user goals into stack-wide configurations [60,142]. RL contributes by learning optimal policies under uncertainty, as the authors in [56] demonstrated with superconducting hardware to improve gate fidelity, an approach extendable to MAC scheduling and transport-layer pacing. Together, these AI paradigms constitute an emerging cognitive control layer essential for maintaining fidelity, optimizing entanglement, and adapting protocol flow in real-time quantum OSI stacks.

### 15.6. Trust and Verification Systems

Establishing trust in quantum networks is inherently complex due to non-deterministic protocols, ephemeral entanglement, and probabilistic security guarantees, prompting the emergence of hybrid architectures combining blockchain, zero-knowledge proofs (ZKPs), and entropy-aware verifiers. Blockchain facilitates tamper-evident logging of QKD sessions, entanglement usage, and inter-node metadata, while ZKPs enable decentralized identity authentication without revealing session content—crucial under the no-cloning constraint [154]. The authors in [155] present privacy-preserving blockchains leveraging ZKPs for secure identity registration and session initiation, enhanced with quantum entropy sources for non-replicable handshake randomness [156]. The authors in [157] extend this vision toward Web3-compliant infrastructures that incorporate lattice-based circuits and entropy verifiers, thereby establishing quantum-safe identity frameworks. Operationally, the authors in [158] demonstrate that QKD key freshness, combined with blockchain-authenticated session history, ensures traceability, privacy, and non-repudiation in entangled communication systems. To organize these security mechanisms under a coherent adversarial framework, we identify five threat categories relevant to the Quantum-Converged OSI stack and map each to its defensive countermeasure and the layer at which that defense is instantiated.

**Physical-Layer Attacks (Layers 0–1):** Photon-number splitting (PNS) attacks exploit multi-photon pulses in weak-coherent QKD implementations; the primary defense is the decoy-state protocol, which randomizes pulse intensities to detect anomalous channel statistics [2]. Side-channel attacks targeting detector blinding or timing jitter are countered by MDI-QKD, which removes all detector-side assumptions from the security proof [100]. A further class of physical-layer vulnerability targets the calibration and synchronization subsystem rather than the quantum channel itself. Dakhkilgova [159] demonstrates that QKD systems are susceptible to nonclassical attacks on the calibration process, which do not affect protocol-level cryptographic strength but expose the synchronization channel to unauthorized access. The proposed countermeasure—attenuating sync pulses to the photon level during time-frame detection—complements MDI-QKD by closing a threat vector outside the scope of the quantum protocol itself. Singh et al. [107] further characterize the synchronization attack surface in fiber-optic QKD, showing that optical emission interception during synchronization represents an independent risk requiring dedicated algorithmic protection at Layer 1.

**Control-Plane Attacks (Layers 2–3):** Quantum denial-of-service (QDoS) attacks flood a node with low-fidelity entanglement requests, exhausting quantum memory; the defense at Layer 2 is telemetry-driven admission control enforcing minimum fidelity thresholds [113]. Routing manipulation attacks inject false fidelity telemetry to divert traffic through compromised nodes; PQC-signed routing updates at Layer 3 constitute the primary countermeasure [144].

**Session and Identity Attacks (Layers 4–5):** Authentication failure in QKD arises when session initiation relies on classical channels vulnerable to impersonation; the defense is identity-bound session negotiation using QKD-derived credentials combined with blockchain-anchored session histories [158]. Entanglement hijacking via false heralding signals is countered by quantum-authenticated handshake protocols at Layer 5 that bind session tokens to entanglement-verified identity assertions [131].

**Dishonest Service Providers (Layers 6–7):** Service providers at the application and presentation layers may misrepresent entanglement fidelity, selectively withhold telemetry, or deliver degraded quantum resources while claiming SLA compliance. The primary defense is entropy-audited service attestation, in which independently verifiable fidelity measurements are logged to a tamper-evident blockchain ledger, enabling third-party auditing of provider claims [157].

**Orchestration-Layer Attacks (Layer 8):** Malicious orchestration agents, whether compromised LLM policy translators or rogue RL routing agents, are defended against via zero-knowledge proof-based policy verification, in which orchestration commands must be accompanied by a verifiable proof of SLA compliance before execution [154]. Control-plane compromise is mitigated by federated orchestration architectures with distributed trust anchors that prevent any single controller from holding unilateral path authority [48].

## 16. Conclusions

The rapid advancement of quantum communication—spanning entanglement distribution, quantum error correction, and programmable interfaces—necessitates a paradigm shift beyond the classical OSI framework. This survey presented a quantum-converged protocol stack incorporating two additional layers: Layer 0 (Quantum Substrate) and Layer 8 (Cognitive Intent Plane), enabling fidelity-aware transport, quantum session abstraction, and LLM-assisted orchestration. Layer-wise analysis identified opportunities for integrating enabling technologies such as QML, RIS, PQC, and hybrid SDQN. Evaluation dimensions included fidelity, entropy throughput, and coherence latency, while tools such as NetSquid, QuNetSim, and QuISP provided simulation-based validation. We identified open research challenges in developing stackless real-time quantum flows, co-evolving QML–LLM agents for orchestration, and creating interoperable standards across hybrid infrastructures. Future directions include quantum-internet-ready stack architectures, cross-layer QEC strategies, SDQN-driven programmability, and standardized performance metrics such as QBER, routing entropy, and teleportation delay. Achieving globally scalable, intelligent quantum internetworks for 7G and beyond demands deep co-design across AI, secure abstractions, and physics-aware networking—laying the foundation for robust, policy-driven, and application-adaptive quantum communication systems.

## Figures and Tables

**Table 1:** Summary of Abbreviations (Part I).

Abbreviation	Definition
ARQ	Automatic Repeat reQuest
BSM	Bell State Measurement
CRC	Cyclic Redundancy Checks
DQML	Distributed Quantum Machine Learning
ETSI	European Telecommunications Standards Institute
EPR	Einstein-Podolsky-Rosen
FEC	Forward Error Correction
FSO	Free-Space Optics
GPT	Generative Pre-trained Transformer
IDQ	ID Quantique
LLM	Large Language Model
MAC	Medium Access Control
MDI	Measurement-Device-Independent
NFV	Network Function Virtualization
NISQ	Noisy Intermediate-Scale Quantum
NV	Nitrogen-Vacancy
OSI	Open Systems Interconnection
PHY	Physical Layer
PICs	Photonic Integrated Circuits
PQC	Post-Quantum Cryptography
QaaS	Quantum-as-a-Service
QAI	Quantum Artificial Intelligence
QASM	Quantum Assembly Language
QBER	Quantum Bit Error Rate
QEC	Quantum Error Correction
QDEs	Quantum Development Environments
QFCs	Quantum Frequency Converters

**Table 2:** Summary of Abbreviations (Part II).

Abbreviation	Definition
QFL	Quantum Federated Learning
QIoT	Quantum-enabled IoT
QIR	Quantum Intermediate Representation
QKD	Quantum Key Distribution
QML	Quantum Machine Learning
QNID	Quantum Network Identifier
QPACE	Quantum Protocol-Aware Cloud Edge
QPU	Quantum Processing Unit
QRNA	Quantum Repeater Network Architecture
QSDs	Quantum Session Descriptors
QSMC	Quantum Secure Multiparty Computation
QSMs	Quantum Session Managers
QSPN	Quantum Source Path Negotiation
RIS	Reconfigurable Intelligent Surface
RL	Reinforcement Learning
SDN	Software-Defined Networking
SDQN	Software-Defined Quantum Networking
SLA	Service-Level Agreement
SLOs	Service-Level Objectives
SLR	Systematic Literature Review
SPDs	Single-Photon Detectors
TaaS	Teleportation-as-a-Service
TCP	Transmission Control Protocol
TTL	Time-to-Live
UAV	Unmanned Aerial Vehicle
ZKP	Zero-Knowledge Proof
ZPL	Zero Phonon Line

**Table 3:** Quality Assessment Score Distribution Across Included Studies (n=167).The aggregate row is set in bold to distinguish the composite score from the five individual dimension scores.

Dimension	Mean Score	Std. Dev.
Relevance	1.84	0.31
Layer Specificity	1.61	0.47
Modeling/Implementation Clarity	1.43	0.52
Innovation	1.55	0.49
Rigor	1.67	0.44
**Aggregate**	**8.10**	**1.38**

**Table 4:** Comparison of Quantum Network Survey Articles. ∘ = not addressed; • = partially addressed; •• = fully addressed with formal treatment. The row labelled “Ours” is set in bold to identify the present survey among the compared works.

Ref.	Quantum	Network	Retran. & Routing	Control	C/Q	Security	Primary Contribution
[28]	••	••	••	••	••	••	Distributed quantum stack
[17]	••	••	∘	••	••	∘	Cross-layer entropy feedback
[29]	∘	••	••	∘	••	∘	QKD routing protocols
[30]	••	••	•	••	••	•	Non-terrestrial quantum networks
[31]	∘	••	∘	∘	••	••	Satellite-based QKD
[32]	∘	•	∘	∘	••	∘	Network softwarization
[33]	••	••	••	••	••	••	AI-enhanced quantum networking
**Ours**	••	••	••	••	••	••	Nine-layer OSI-based quantum networking framework

**Table 5:** Projects and Research Addressing Quantum Networking Challenges.

Quantum Challenge	Projects/Protocols	Research
Quantum Stack Architecture	SEQUOIA, Quantum Internet Alliance, EU Quantum Flagship	[1,27]
Entanglement Distribution	QuNetSim, NetSquid, QRNA, QLoop	[5,50]
Retransmission and Quantum Routing	QSPN, Q-RoadNet, QStackSim	[1,5]
Control Plane and Stack Orchestration	ETSI ISG QKD, QuantumIRIS, LUMIQ-NET	[1,2]
Hybrid Classical/Quantum Integration	QKDnet, Q3P, QPACE	[2,5]
Quantum Security (QKD, PQC)	SECOQC, OpenQKD, IDQ Network	[1,2]
AI/QML-enhanced Network Intelligence	QML-Stack, QuantumGraphNet	[5,50]

**Table 6:** Layer-by-layer definition of the proposed Quantum-Converged OSI stack.

Layer	Description
8	Cognitive Intent Plane—LLM-based orchestration
7	Application—Quantum APIs and overlay systems
6	Presentation—Quantum-native metadata formatting
5	Session—Contextual handshake with quantum tokens
4	Transport—Flow entropy, PQC reliability
3	Network—Entanglement routing, SDN control
2	Data Link—Fidelity-aware MAC, QEC
1	Physical—RIS-enabled entangled channels
0	Quantum Substrate—Decoherence and teleportation

**Table 7:** Quantum Network Stack Elements and Technologies. Entries in the first column are set in bold because they act as row headers.

Quantum Stack	Sample Technologies/Concepts
**Entanglement**	EPR Pair IDs, Bell Pair Tracking, Quantum State Hashing
**Quantum Address**	Quantum Network Identifiers (QNID), Entangled Node ID, Quantum MAC Address
**Quantum Sensing**	Quantum-enhanced sensors, Single-photon detectors, Quantum radar, Spin-based magnetometers [65,66,67]
**Quantum Networks**	QKD, Teleportation Protocols, BB84, E91, MDI-QKD, QRNA, QLoop, Qubus Protocols [2,31,46]
**Quantum Computation**	
**Hardware**	IonQ, IBM Q, Rigetti, PsiQuantum, Photonic Chips, Quantum Repeaters, Quantum Routers [20,58,68]
**Software**	Qiskit, Cirq, QuNetSim, NetSquid, QuISP, Strawberry Fields, QCoDeS, ProjectQ [14,69,70,71]
**Quantum Services**	Entanglement-as-a-Service (EaaS), Quantum Key-as-a-Service (KaaS), Quantum Cloud API, Quantum Orchestration Services, LLM-Aided Stack Automation [60,72]
**Quantum Semantics**	Quantum Context Encoding, Entanglement Metadata, Fidelity Metrics, Entropy Cost, Stack-Level Intent Modeling [17,54,73]

**Table 8:** Inter-layer interface primitives in the Quantum-Converged OSI stack. Upward primitives export telemetry to higher layers; downward primitives deliver configuration or control commands to lower layers.

Boundary	Upward Primitive	Downward Primitive
L0 → L1	entanglement_state(fidelity, $T_{2}$)	schedule_entanglement(node_id)
L1 → L2	link_telemetry(QBER, jitter, loss)	set_encoding(modality)
L2 → L3	memory_state(age, freshness, QEC_status)	admit_entanglement(threshold)
L3 → L4	path_score(fidelity, hops, entropy)	reroute(path_id)
L4 → L5	session_viability(coherence_budget)	flush_qubits(session_id)
L5 → L6	role_binding(identity, scope)	set_encoding_profile(task)
L6 → L7	semantic_descriptor(format, fidelity)	transcode(target_platform)
L7 → L8	intent(fidelity_SLA, latency_bound)	reconfigure_stack(policy)

**Table 9:** Comparison of selected quantum substrate platforms across coherence, fidelity, and scalability.

Platform	Coherence Time	Fidelity	Entanglement Range	Scalability	Ref.
Trapped Ions	>1 s	High	Local–Regional	Medium	[77]
NV Centers	ms	Medium–High	Metropolitan	Medium	[78]
SPDC Photonics	ns–μs	Medium	Long-distance (fiber/satellite)	High	[79,80]

**Table 10:** Comparison of multipartite entangled resource states for the Layer 0 quantum substrate.

State	Loss Tolerance	Generation	Key Platform	Ref.
GHZ	None (fully fragile)	Det./Prob.	Photonic, Ion trap	[88]
W (=$D_{1}^{n}$)	High (partial entanglement persists)	Det. (ions), Prob. (photonic)	Ion trap, NV center, Photonic	[88,89,90]
Dicke $D_{k}^{n}$	Moderate (symmetric noise robust)	Det. (circuit), Prob. (photonic)	Photonic, Superconducting	[94,95]

**Table 11:** Characteristics of Quantum PHY Protocols.

Quantum PHY Protocol	Qubit Medium	Encoding Technique	Operating Band	Error-Control Method
BB84 (Fiber) [2,46]	Photons	Polarization Encoding	1550 nm (C-band)	Decoy State + Error Estimation
E91 [46]	Photons	Entangled Pairs	1310/1550 nm	Bell Test Verification
MDI-QKD [100]	Photons	Time-bin or Phase	1550 nm	BSM
Qubits over NV Centers [65,66]	Electron Spins	Spin-Based Optical	637 nm (ZPL)	QEC
Continuous-Variable QKD [46]	Coherent States	Gaussian Modulation	1550 nm	Reconciliation + Homodyne Detection
Satellite QKD (Micius) [31,86]	Photons (Space)	Polarization	Free-Space Optical	Adaptive Optics + Post-selection
Quantum Teleportation [46,65]	Entangled Photons	Dual-Rail	1310/1550 nm	Bell Measurement + Feedforward

**Table 12:** Comparison of quantum transmission media for Layer 1 physical-layer implementations.

Medium	Distance	Decoherence	Env Sensitivity	Scalability	Examples
Optical Fiber	100 km	Medium	Low	High	Fiber-based QKD
Free-Space Optics	100–1000 km	High	Very High	Medium	Satellite–Ground Links
On-Chip PIC	cm–m	Low	Low	Very High	Quantum Routers
RIS-Enhanced Channels	100 m–km	Adaptive	Moderate	Experimental	Smart Indoor Quantum Links

**Table 13:** Comparison of selected MAC and QEC strategies for Layer 2 implementation.

Protocol	Type	Strengths	Limitations	Ref.
Q-MAC	Probabilistic	Entanglement-aware, Fidelity-driven	Local scope only	[114]
Adaptive MAC	Dynamic	Real-time reallocation, Memory-sensitive	High control complexity	[117]
Surface Code	Topological	High fault tolerance	Requires many ancilla	[39]
Modular QEC	Localized	Hardware-adaptive, Minimal overhead	Platform-specific tuning	[40]

**Table 14:** Comparison of Layer 3 routing protocols in quantum networks.

Protocol	Type	Strengths	Limitations	Ref.
SDQN	Centralized	Policy-based, telemetry-driven	Control latency	[48]
Bell-Chain Probabilistic	Stochastic	Coherence-aware path modeling	Fragile swap dependencies	[120]
Deep RL Routing	AI-Based	Adaptive to real-time fidelity drift	Convergence time	[122]
Hybrid RL-Heuristic	Combined	Balance speed and precision	Requires policy tuning	[123]

**Table 15:** Comparison of quantum transport-layer strategies and their capabilities.

Protocol/Feature	Objective	Limitation	Ref.
SessionQ	Session abstraction	Path rebind complexity	[127]
QTransport Contract	SLA enforcement	Requires deep telemetry	[19]
Entanglement Budgeting	Qubit scheduling	SLA enforcement overhead	[113]
Fidelity-Aware Flow Control	Session integrity	Irreversible losses on error	[121]
Multiplexing	Concurrent sessions	Flow interference, tagging	[130]

**Table 16:** Session-layer role-binding models and their operational characteristics.

Model	Use Case	Identity Binding	Scope	Ref.
Teleportation Triad	TaaS, Circuit Injection	Yes	Node-local	[132]
QKD Bidirectional	Secure Key Setup	Yes	Pairwise Link	[135]
Stateless Session	Blind Quantum Compute	Optional	Single-flow	[136]
Federated Mapping	DQML, Entangled Voting	Yes	Distributed Role-Aware	[131]

**Table 17:** Quantum encoding schemes used at the presentation layer across hardware platforms.

Encoding	Platform	Fidelity	Transformability	Use Cases	Ref.
Polarization	Photonic	Medium	High	QKD, DQS, QVote	[137]
Spin-based	NV/Ion Trap	High	Low	Memory, Logic Circuits	[24]
Orbital Angular Momentum	FSO/Photonic	High	Low	Satellite	[139]
Dual-Rail	Photonic	Medium	High	Short-range QKD, Sensors	[138]

**Table 18:** Quantum application classes and their operational parameters at the application layer.

Application	Fidelity	Timing	Role Binding	API Primitive	Ref.
QKD	High	Medium	Yes	initQKDSession()	[135]
Teleportation Service	Very High	High	Yes	requestTeleportation()	[132]
DQML	Medium–High	Tight	Yes	submitQuantumCircuit()	[136]
Quantum Sensing	Medium	Loose	Optional	startSensorStream()	[13]
Quantum Consensus	High	Real-time	Yes	initConsensusTask()	[131]

**Table 19:** Orchestration and cognitive control functions at Layer 8.

Function	Description	Affected Layers	Ref.
Intent Compilation	Translate application-level SLAs into stack configurations	L1–L7	[141]
Closed-loop Reconfiguration	Dynamic policy updates from telemetry triggers	L1–L5 ⇔ L8	[113]
Cognitive Control (AI)	Learn fidelity patterns and predict service degradation	L2–L5	[136]
Trust Domain Enforcement	Map identity to entanglement policy domains	L5, L7	[132]
SLA Enforcement	Guarantee application-level entropy/fidelity contracts	L4–L7	[121]
Declarative API Interface	Expose orchestrator control as programmable service descriptors	L8 Admin	[48]

**Table 20:** Cross-layer functional dependencies in quantum networks.

Cross-Layer Theme	Layers Involved	Description
Telemetry Propagation	L2–L8	Vertical QBER, coherence, routing state sharing [113]
Intent Compilation	L7 → L1–L6	Service goal decomposition and reconfiguration [141]
Trust Mapping	L5 → L2–L3	Identity propagation and domain exclusion [131]
Fidelity-Aware Pacing	L2–L4–L8	Session, memory, transport tuning based on fidelity [121]
Role-Aware Routing	L5–L3	Entanglement routing based on session logic [132]
Semantic Encoding Tuning	L6–L8	Format tuning by app context and fidelity goals [24]

## Data Availability

No new data were created or analysed in this study. Data sharing is not applicable to this article.
